# Autonomic Nervous System Function in Anorexia Nervosa: A Systematic Review

**DOI:** 10.3389/fnins.2021.682208

**Published:** 2021-06-28

**Authors:** Zoe M. Jenkins, Nina Eikelis, Andrea Phillipou, David J. Castle, Helen E. Wilding, Elisabeth A. Lambert

**Affiliations:** ^1^Iverson Health Innovation Research Institute, Swinburne University of Technology, Melbourne, VIC, Australia; ^2^Department of Mental Health, St Vincent's Hospital Melbourne, Fitzroy, VIC, Australia; ^3^Department of Psychiatry, University of Melbourne, Melbourne, VIC, Australia; ^4^Centre for Mental Health, Swinburne University of Technology, Melbourne, VIC, Australia; ^5^Department of Mental Health, Austin Health, Melbourne, VIC, Australia; ^6^Library Service, St Vincent's Hospital Melbourne, Fitzroy, VIC, Australia

**Keywords:** anorexia nervosa, eating disorders, autonomic nervous system, sympathetic nervous system, parasympathetic nervous system, heartrate variability, noradrenaline, orthostatic response

## Abstract

**Background:** Autonomic nervous system (ANS) dysfunction has been suggested to contribute to the high prevalence of cardiovascular complications in individuals with anorexia nervosa (AN), yet has not been thoroughly investigated. The current review aimed to synthesize the evidence of basal ANS function in individuals with a current diagnosis of AN and those with a previous diagnosis who had achieved weight restoration, as compared to controls.

**Methods:** A systematic review of nine databases was conducted and studies that were published in a peer-review journal, in English, that included at least one assessment of ANS function in individuals with a current or previous diagnosis of AN were selected. Forty-six studies were included with a total of 811 participants with a current diagnosis of AN and 123 participants with a previous diagnosis of AN.

**Results:** ANS function was assessed through heart rate variability (*n* = 27), orthostatic challenge, blood pressure variability or baroreflex sensitivity (*n* = 11), adrenergic activity (*n* = 14), skin conductance level (*n* = 4), and pupillometry (*n* = 1). Individuals with AN demonstrated increased parasympathetic activity and decreased sympathetic activity, suggestive of autonomic dysregulation. Following weight restoration, autonomic function trended toward, or was equivalent to, control levels.

**Discussion:** Autonomic dysregulation is indicated through a range of assessments in individuals with AN. Future investigations should utilize a variety of assessments together in order to conclusively establish the nature of autonomic dysfunction in AN, and following extended weight restoration. Moreover, investigation into the co-occurrence of ANS function and cardiovascular risk is required.

## Introduction

Anorexia Nervosa (AN) is an eating disorder characterized by restriction of food intake, an intense fear of gaining weight and a distorted self-perception of body image (American Psychiatric Association, 2013). AN has been recognized as an increasingly prevalent psychiatric condition among young people in Western societies, with the incidence also increasing in a variety of racial and ethnic groups (Nakai et al., 2016), mostly in women (Hoek, 2006). AN has a typical onset in adolescence (Hoek and Van Hoeken, 2003) and has an estimated lifetime prevalence of 1.7% in the general population (Smink et al., 2014). The etiology and pathophysiology of AN are complex, involving biological, psychological, and sociocultural development and maintenance factors (Phillipou et al., 2019). The chronic nature of AN is evidenced by a 50% relapse rate (Pike, 1998), with learned maladaptive behaviors becoming deeply entrenched and difficult to alter (Steinglass and Walsh, 2016).

The energy deprivation and malnutrition associated with AN places immense pressure on the cardiovascular system, with up to 80% of patients suffering from cardiovascular complications (Spaulding-Barclay et al., 2016). These include structural, conduction, and hemodynamic abnormalities (Sachs et al., 2016; Giovinazzo et al., 2019; Smythe et al., 2020), and are a major contributor to the high mortality rate in AN (Nakai et al., 2016), which is approximately six times that of the general population (Papadopoulos et al., 2009; Arcelus et al., 2011). Cardiovascular problems occur not only during the starvation state of AN; there are also specific cardiac complications that arise during the process of re-feeding, such as arrhythmia, tachycardia, and congestive heart failure (Casiero and Frishman, 2006; Vignaud et al., 2010). Despite the profound psychological and physical burdens that accompany AN, the underlying physiological mechanisms behind the cardiovascular complications of the illness remain poorly understood. It has been suggested that disturbances in cardiac autonomic regulation may contribute to the increased cardiovascular complications and mortality in AN (Mazurak et al., 2011a).

The autonomic nervous system (ANS) provides the link between the cardiovascular system and the central nervous system, and is responsible for the regulation of internal bodily processes in response to physiological and environmental changes (Palma and Benarroch, 2014). The ANS is a dynamic regulatory function that involves interpretation of sensory feedback from the organs by higher brain areas, including the brainstem and hypothalamus, in order to adapt the output of the ANS to adjust the physiological state of the body (Porges, 2007; Buijs et al., 2013). Through the regulation of heart rate (HR), blood pressure (BP), and rate of respiration among other visceral activities, the ANS maintains cardiovascular homeostasis via the opposing inputs of its two branches; the sympathetic (SNS) and parasympathetic (PNS) nervous systems (Gordan et al., 2015). Activation of the SNS results in increased arousal, such as increased HR and blood vessel constriction through the release of noradrenaline (NE), whereas the PNS (or vagal nerve) acts in opposition to decrease HR and BP. Evaluation of the ANS can be derived from various techniques including hemodynamic, biochemical and neurophysiological assessments with each presenting its own limitations (Grassi and Esler, 1999). Therefore, multiple assessments of autonomic function should be undertaken together in order to provide an overview of neural function; some of which are briefly detailed below.

Hemodynamic assessments can provide insight into the autonomic regulation of blood flow. Sinus bradycardia (Yahalom et al., 2013) and low BP levels (Sachs et al., 2016) are commonly observed in individuals with AN and are suggestive of abnormalities in autonomic regulation of HR and BP. The majority of previous investigations into autonomic function in individuals with AN have assessed heartrate variability [HRV; the beat-to-beat variation in HR (Task Force of The European Society of Cardiology The North American Society of Pacing Electrophysiology, 1996; Billman, 2011)] as an estimation of autonomic cardiac regulation, with inconclusive findings (see Mazurak et al., 2011a for a review). While the review by Mazurak et al. (2011a) found the majority of studies that investigated HRV in AN reported parasympathetic dominance, some reported sympathetic dominance, while others found no difference in comparison to controls; this led the authors to suggest that HRV may not be suitable for the assessment of ANS in AN (Mazurak et al., 2011a). Another hemodynamic assessment of autonomic function is the orthostatic stress test, which provides a window into autonomic regulation through the baroreceptor reflex control of BP and HR (Grassi and Esler, 1999; Westerhof et al., 2006). Conditions related to orthostatic intolerance, such as orthostatic hypotension, syncope and postural orthostatic tachycardia syndrome (POTS) represent autonomic failure and have also been reported in AN (Sachs et al., 2016).

Biochemical assessment of plasma NE levels can provide an index of sympathetic neural function that have been shown to vary according to weight (Lambert et al., 2007). However, circulating NE represents only a fraction of the amount secreted from nerve terminals and is dependent on secretion, clearance and re-uptake processes (Esler et al., 1990), therefore this method provides a “confounded” index of systematic sympathetic activation (Grassi et al., 2015). Measurement of the NE metabolite, 3-methyl-4-hydroxyphenylglycol (MHPG) is another common biochemical assessment that is undertaken to further inform regional NE synthesis, release and re-uptake (Grassi and Esler, 1999).

In addition to regional NE spillover, the other “preferred” assessment for sympathetic nervous system evaluation, is the neurophysiological technique of “microneurography” (Grassi et al., 2015). Microneurography provides a direct continuous recording of muscle sympathetic nerve activity (MSNA) to give a measure of central nervous system sympathetic neural outflow to the skeletal muscles (Grassi and Esler, 1999), including blood vessels. Increased sympathetic neural drive, as assessed by microneurography, is associated with increased cardiovascular risk (Kaye et al., 1995; Grassi, 2006), yet microneurography remains less commonly used due to its semi-invasive nature.

It is beyond the scope of the current review to provide an overview of all assessments of ANS function; previous thorough reviews have been conducted (Grassi and Esler, 1999; Tjalf and Timo, 2019). To our knowledge, there has been no prior systematic review of autonomic function in individuals with AN. Moreover, most studies have primarily assessed function in individuals in the acute state of AN and it is less clear whether any abnormalities persist after weight restoration. In order to advance the knowledge of ANS function in AN, the current systematic review aims to synthesize studies investigating resting-state ANS function in individuals with AN, including those who have achieved weight restoration, as compared to healthy controls. Given the important clinical implications of abnormalities in autonomic cardiovascular control, a greater understanding of any abnormalities in ANS function in individuals with AN, and following weight restoration, is crucial.

## Materials and Methods

### Search Strategy

This systematic review was carried out in accordance with the Preferred Reporting Items for Systematic Review and Meta-Analyses (PRISMA) (Supplementary Material) (Moher et al., 2010) and was registered with the International Prospective Register of Systematic Reviews (PROSPERO identifier CRD42020177195). Studies were identified through systematic searches of nine databases: Ovid MEDLINE(R) ALL 1946 to November 03, 2020; Embase 1974 to 2020 November 03 (Ovid); Ovid Emcare 1995 to 2020 Week 44; APA PsycInfo 1806 to October Week 4 2020 (Ovid); Ovid Nursing Database 1946 to October Week 4 2020; CINAHL (EBSCOhost); Health Collection, Humanities & Social Sciences Collection (Informit); Cochrane Library and Clinicaltrials.gov. Search strategies were developed by a medical librarian, HW, in consultation with the review team. Strategies combined the general concepts of anorexia nervosa AND autonomic nervous system using a combination of subject headings and textwords relevant to each database. Results were limited to English language, but no date limits were applied. Animal studies were excluded. An initial strategy was developed for Medline and then adapted for other databases (Appendix 1 in Supplementary Material). All searches were updated on 5 November 2020. Reference lists of included studies were screened for additional publications.

### Study Selection

Search results were exported to Endnote bibliographic management software, duplicates removed, and the remainder uploaded to Covidence systematic review software (www.covidence.org) by HW. Two authors (Z.J., E.L.) independently screened records on title and abstract and then full text against the following exclusion criteria: primary condition not AN, no diagnostic criteria referenced, no control group, no basal ANS assessment outcome, protocol paper, review article, dissertation, conference abstract, case series/study. A third reviewer (N.E.) resolved any conflicts. Studies that included at least one of the ANS measures in basal conditions listed in Table 1 were included (see Table 1 for a summary of ANS outcomes, description of assessment and relationship to ANS functioning). A meta-analysis was not performed as there were too few similarities between study methods and measures.

**Table 1:** Description of ANS outcomes included in the review.

**Domain**	**Measure**	**Acronym**	**Significance and methodology**	**Parameters**	**ANS indicator**	**Number of included studies using the measure**
Heart rate variability (HRV)	Standard deviation of normal-to-normal intervals	SDNN or R-R-interval-SD	Measured by calculating the average change in the duration of the interval (in ms) between consecutive heart-beats (R-R intervals) and deriving the standard deviation of the N-N (or R-R) intervals over a time section of the ECG.	SDNN	Higher SDNN: increased HRV: increased PNS functioning: hypo-arousal	27 (any measure of HRV)
	Root Mean Square of Successive Differences (HRV)	RMSSD	A measure of HRV which is measured as the average root square of the interval between successive peaks in ECG. Compared to SDNN, it is considered more reliable in measuring HRV.	RMSSD	Higher RMSSD: increased HRV: higher PNS functioning: hypo-arousal	
	Normal-to-normal intervals > 50 ms (% of)	NN50 (pNN50)	Measured on ECG as the number of pairs of successive IBI intervals which are different by 50 ms or more. pNN50 is the proportion of NN50, in relation to the entire number of RR intervals.	Number of NN50	Higher number of NN50 (or higher pNN50): increased HRV: higher PNS functioning: hypo-arousal	
	Low frequency power	LF	As a frequency domain measure, it represents the amount of spectral power between 0.04 and 0.15 Hz on the Fast Fourier Transform (FFT) spectrum of HRV. It is indicative of the baroreflex which is a modulation (acceleration or deceleration) of HR in situations when blood pressure (BP) is too low or high (respectively), with the aim of changing BP levels through modulating HR.	LF power	Increased LF power: increased baroreflex effect: increased HRV	
	High frequency power	HF	Similarly to LF, it is a frequency domain measure of HRV which analyses activity in the 0.15–0.40 Hz range. It has been linked to cardiac-vagal activity, representing parasympathetic modulation of arousal.	HF power	Increased HF power: increased PNS functioning: hypo-arousal	
	Low/high frequency power	LF/HF	The ratio between spectral power in the low and high Frequency range (see above for specific ranges in Hz). It has been used as an index of the balance between SNS and PNS functioning (sympathovagal balance).	LF/HF ratio	Increased LF/HF ratio: sympathetic dominance. Reduced LF/HF ratio: parasympathetic dominance (however a strong debate is going on in literature, regarding the association between LF/HF ratio and ANS)	
	Short-term fractal scaling exponent	α	Using detrended fluctuation analysis, the fractal scaling exponent provides a measure of complexity in heart period series (RR interval) (Peng et al., 1995).	Fractal correlation	Reduced α has been demonstrated in patients with congestive heart failure and depressed left ventricular function	
Baroreflex function	Baroreflex sensitivity	BRS	Invasive: measuring the change in heart rate in response to changes in blood pressure induced by injection of vasoactive drugs that have minimal effect on the sinus node. Non-invasive: the Valsalva maneuver, head-up-tilt, the neck chamber technique (which provides a selective manipulation of carotid baroreceptors), and the analysis of spontaneous variations of blood pressure and RR interval. Consecutive systolic pressure values and corresponding RR intervals with one-beat delay are fitted by a linear regression in the interval between the beginning and end of systolic pressure increase, the sensitivity of the baroreflex is provided by the slope of the fitted line, and expressed as the change in RR interval in milliseconds per millimeter of mercury change in systolic pressure.	ms/mmHg	CV diseases are often accompanied by an impairment of BRS mechanisms, with a reduction of inhibitory activity and an imbalance in the physiological sympathetic-vagal outflow to the heart, thus resulting in a chronic adrenergic activation. Sustained baroreflex-mediated increase in sympathetic activity may contribute to increased end-organ damage and to the progression of the underlying disease, and a blunted baroreflex gain is predictive of increased cardiovascular risk in post-myocardial infarction and heart failure patients.	4
Blood pressure	BP response to orthostatic challenge	BP response	Orthostatic maneuvers are commonly used as a test procedure to assess autonomic nervous system control of the circulation. The physiological response to orthostatic stress is assessed by observing the difference in systolic blood pressure (SBP) and diastolic blood pressure (DBP) during a change from horizontal to vertical body position.	SBP, DBP	Alterations in the autonomic nervous system may contribute to an increase or decrease in resting blood pressure response to an orthostatic challenge.	7
	Blood pressure variability	BPV	Ultrashort-term: direct continuous intra-arterial recordings coupled to spectral analysis; short-term: direct continuous intra-arterial recordings, ABPM; long-term: office blood pressure, ABPM, home blood pressure monitoring.	Ultrashort-term (very low frequency, low frequency and high frequency BPV); beat-to-beat variation. Short term BPV; minutes to hours. Long-term BPV: day-to-day, visit-to-visit.	Ultrashort-term: estimation of neurohumoral systems involved in BP regulation; short-term: increased variability in daytime, night-time, and whole 24 h period associated with increased target organ damage; long-term: large visit-to-visit BPV independently associated with increased incidence of stroke.	3
Catecholamines	Noradrenaline/Norepinephrine	NE	Urine: activity of the adrenergic nervous system is inferred from 24 h urinary excretion of noradrenaline, adrenaline and their precursors or metabolites. Plasma: measurement of plasma noradrenaline concentration in venous blood.	Urine: excretion of noradrenaline, adrenaline and their precursors or metabolites over the past 24 h. Plasma: noradrenaline levels pg/ml.	Activity of the adrenergic nervous system: increased activity indicates increased sympathetic arousal	14
	3-Methoxy-4-hydroxyphenylglycol	MHPG	MHPG is a major metabolite of NE and reflects noradrenergic neuronal tone in humans	Urine: excretion of MHPG over the past 24 h. Plasma: MHPG levels pg/ml.	Increased levels indicate increased activity of the adrenergic nervous system: increased activity indicates increased sympathetic arousal	4
Electrodermal activity (EDA)	Skin conductance level	SCL	An electrical potential is applied to two electrodes, placed next to each other on the hand palms. The electrical current, flowing between the electrodes, is measured as an index of skin conductance.	Mean SCL, Change (slope) of SCL over time	Higher SCL: increased sympathetic arousal	4
Pupillometry	Pupil diameter	PLR	Pupil size or diameter is measured using eye-tracking or optometrist tools, carefully considering for any confounding effects, including changes in environmental luminance or body movements. Pupil constrictions are generally associated with increase of brightness and processing of visual information situated at a close distance (due to narrowing of visual attentional focus).	Mean pupil size (tonic) Stimulus-or event-locked changes in pupil size (phasic)	Increased tonic pupil size: increased allocation of mental resources to the external environment. Phasic pupil size dilations: increased arousal, spontaneous or in response to a stimulus	1

### Data Extraction

Two reviewers (Z.J. and E.L.) independently extracted data and consensus was confirmed by a third reviewer (N.E. or A.P.) Extracted data included information on study characteristics and basal ANS assessment and outcomes.

### Risk of Bias/Quality Assessment

The risk of bias among included studies was assessed independently by two authors (Z.J. and D.C.) using a modified version of the Newcastle-Ottawa Quality Assessment Scale (NOS; see Appendix 2 in Supplementary Material) for cohort/case-control studies, in which a high score indicates a low risk of bias (Wells et al., 2006). Studies were assessed on three domains; participant selection, comparability and outcome assessment and were classified as at low, moderate, or high risk of bias. The risk of bias was not used as an exclusion criterion in the selection of studies to provide a complete overview of available data.

### HRV Risk of Bias/Quality Assessment

Given the large number of included studies that assessed HRV, we used a modified version of a previously published measure of study quality in studies of HRV in functional somatic disorders to specifically evaluate quality of HRV methods (Tak et al., 2009). We modified the tool to incorporate the items listed in the Guidelines for Reporting Articles on Psychiatry and Heart rate variability (GRAPH) criteria (Quintana et al., 2016) to provide a more comprehensive assessment of HRV quality and risk of bias (see Appendix 3 in Supplementary Material). We assessed three general domains: appropriate selection of participants, appropriate quantification collection of HRV and appropriate control for confounding factors. Potential scores ranged from 0 to 22.

## Results

**Figure 1:**
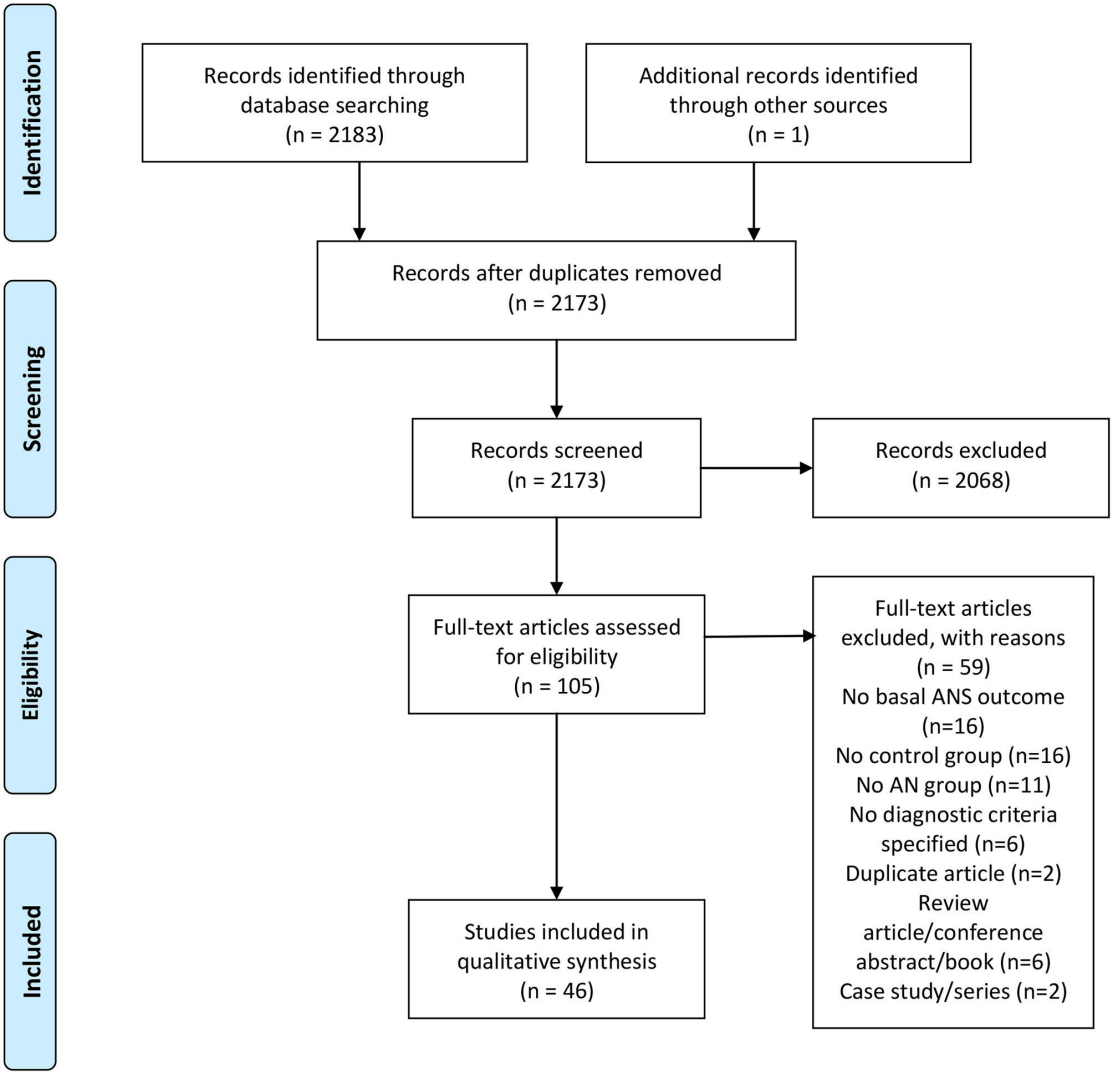
PRISMA flowchart. From Moher et al. (2009).

The literature search yielded 2,126 unique citations. The full text of 105 citations were examined and, of these, 46 articles met our inclusion criteria (see Figure 1).

### Study Characteristics

Characteristics of the included studies for qualitative synthesis are shown in Table 2. All included studies utilized cross-sectional study design; 39 assessed participants at a single time point and seven included assessments at multiple time points after weight restoration (Gross et al., 1979; Riederer et al., 1982; Lesem et al., 1989; Kaye et al., 1990; Kreipe et al., 1994; Bar et al., 2006; Lachish et al., 2009). The 46 studies included assessments of 811 participants with a current diagnosis of AN (757 female, 11 male, 43 not specified), 123 participants with a previous diagnosis of AN who were at various stages of treatment and weight restoration (AN-WR; 100 female, 2 male, 21 not specified) and 867 control participants (834 female, 20 male, 13 not specified). Sample sizes ranged from 7 to 89 participants with a current diagnosis of AN, 4–18 weight-restored participants, and 8–39 controls. One study did not specify the sample size of their control group (Lechin et al., 2010), four studies did not specify the sex of the AN participants (Kaye et al., 1990; Pirke et al., 1992; Rommel et al., 2015; Palomba et al., 2017) and three studies did not specify the sex of the AN-WR participants (Riederer et al., 1982; Kaye et al., 1990; Pirke et al., 1992). The average duration of illness ranged from 8 months to 10 years and the duration of weight restoration ranged from 2 weeks to 3 years.

**Table 2:** Characteristics of included studies.

**References**	**Group**	**Sample size, sex**	**Age (years)**	**BMI (kg/m^**2**^)**	**Duration of AN/WR**	**AN subtype**	**Inclusion criteria**	**Exclusion criteria**	**Criteria used to classify AN**	**Patient setting**	**Medication**	**Comorbid psychiatric diagnoses**	**Basal ANS variables assessed**	**Risk** **of bias**
Abell et al. (1987)	AN	6F, 2M	Mean: 20	NS	27.75 months	6 AN-R, 2 AN-BP	Met criteria for AN	NS	DSM-III	NS	NS	NS	SCL	Low
	HC	6F, 2M	NS	NS			Age- and sex-matched to AN group, no history of recent dieting, weight or exercise change. Normal thyroid function.							
Bar et al. (2006)	AN (T1)	15F	16.1 ± 0.5	15.1 ± 0.5	8.6 ± 4 months	NS	Met criteria for AN	History of peripheral neuropathies, cardiac arrhythmia, or alcohol or drug abuse	DSM-IV	Inpatient	Excluded	Excluded	HRV (5 min), PLR	Low
	AN-WR (T2)	15F	NS	NS	0 months	NS	Previously met criteria for AN, after reaching 25th percentile of normal weight							
	AN-WR (T3)	15F	NS	NS	6 months	NS	Previously met criteria for AN, 6-months after reaching 25th percentile of normal weight							
	HC	15F	16.6 ± 0.5	21.4 ± 0.9			Age-, sex, handedness- and education-matched to AN group							
Bartak et al. (2004)	AN	10F	23.0 ± 1.2	15.6 ± 0.6	NS	NS	Met criteria for AN, non-smokers, no allergies and no medication for at least 1 month before the study	Professional athletes	DSM-IV	Inpatient	Excluded	NS	NE (plasma)	Low
	HC	10F	23.3 ± 1.0	21.6 ± 0.4			Age- and sex-matched to AN group, non-smokers, no allergies and no medication for at least 1 month before the study	History of obesity or malnutrition, CV disease, ED or other psychiatric disorders, abnormal physical examination and ECG						
Billeci et al. (2015)	AN	27F	14.6 ± 2.2	15.7 ± 2.1	18 ± 14 months	All AN-R	Met criteria for AN-R subtype, Wechsler Full Scale IQ > 80	Psychotic symptoms, comorbid conditions not related to eating disorders, substance abuse, AN-BP subtype	DSM-IV and DSM-5	Inpatient	NS	AN: 59.2% MDD; Dysthymic disorder: 37%: GAD: 11.11%; ODD: 3.7%	HRV (15 min)	Low
	HC	15F	14 ± 1.5	20.5 ± 2.2			Sex-matched to AN group, Wechsler Full Scale IQ > 80	NS						
Billeci et al. (2019)	AN	23F	15.2 ± 1.9	15.7 ± 1.6	19.1 ± 14.5 months	All AN-R	Met DSM-IV and DSM-5 criteria for AN-R subtype, Wechsler Full Scale IQ > 80, within 3 days of hospitalization	Psychotic symptoms, comorbid conditions not related to eating disorders, current or previous episodes of substance abuse, AN-BP subtype	DSM-IV and DSM-5	Inpatient	Excluded	NS	HRV (5 min)	Low
	HC	17F	15.7 ± 2.1	21.7 ± 2.8			Sex-matched to AN group, Wechsler Full Scale IQ > 80	NS						
Bomba et al. (2014)	AN	21F	15.9 ± 1.1	15.1 ± 2.6	NS	All AN-R	Met criteria for AN-R subtype	Drug or alcohol use	DSM-IV	NS	NS	NS	HRV (24-h)	Low
	HC	21F	16 ± 2.0	19.7 ± 1.8			Age- and sex-matched to AN group, normal BMI, regular ovulatory menstrual cycles	Neurological or psychiatric disorders, thyroid diseases, drug or alcohol use, androgenic symptoms, steroid hormone use						
Calloway et al. (1983)	AN-R	12F	20.3 ± 6.7	NS	4.1 ± 3.7 years	12 AN-R	Met criteria for AN	NS	Russell's criteria (Russell, 1970)	NS	NS	NS	SCL	Moderate
	AN-BP	10F	21.8 ± 4.3	NS	4.9 ± 3.3 years	10 AN-BP								
	HC	32F	Mean: 23.5	NS			Sex-matched to AN group							
Casu et al. (2002)	AN	13F	25.0 ± 5.8	16.9 ± 2.6	Range of 3–7 years	NS	Met criteria for AN	Taking medication known to alter blood pressure or heart rate	DSM-IV	NS	NS	NS	HRV (5 min) and Orthostatic response	Moderate
	HC	16F	25.0 ± 2.3	20.9 ± 0.9			Sex-matched to AN group, normal diet and levels of physical activity							
D'Andrea et al. (2008)	AN	89F	26.2 ± 8.1	14.9 ± 2.5	NS	NS	Met criteria for AN, BMI <17.5	NS	DSM-IV	NS	*N* = 38 (42.7%) taking antidepressants; *N* = 9 (10.1%) taking antipsychotics; *N* = 26 (29.2%) taking benzodiazepines	NS	NE (plasma)	Moderate
	HC	27F	27.7 ± 6.3	NS			Age- and sex-matched to AN group							
De Rosa et al. (1983)	AN	20F, 3M	Mean (range): 22 (15–40)	NS	2.7 ± 2.3 years	NS	Met criteria for AN, 30% below IBW	NS	Halmi's criteria (Halmi et al., 1977)	Inpatient	Suspended for at least one month	NS	NE (urinary)	High
	HC	10F, 5M	Mean (range): 25 (18–45)	NS			NS				None taking pharmaceutical therapy			
Dostalova et al. (2007)	AN	10F	22.1 ± 1.0	15.7 ± 0.5	NS	All AN-R	Met criteria for AN, non-smokers, no allergies, free of medications for 3 weeks prior to study	Professional athletes	DSM-IV	Inpatient	None	NS	NE (plasma)	Low
	HC	15F	21.3 ± 0.9	21.2 ± 0.4			Age- and sex-matched to AN group, non-smokers, no allergies, free of medications for 3 weeks prior to study	History of obesity, malnutrition, CV disease, psychiatric disorders, abnormal ECG						
Galetta et al. (2003)	AN	25F	17.5 ± 4.2	15.3 ± 1.4	2.6 ± 1.8 years	NS	Met criteria for AN, stable BW for 3 months, no signs of CV disease	NS	DSM-IV	Referred for treatment	None	NS	HRV (24-h)	Low
	HC-T	25F	17.7 ± 3.9	18.7 ± 1.7			Age-and sex-matched to AN group, BMI <20	Family history of arterial hypertension						
	HC-NW	25F	18.1 ± 4.5	21.9 ± 2.8			Age- and sex-matched to AN group, BMI>20							
Green et al. (2020)	AN	7F	NS	18.5 ± 1.7	NS	NS	Met criteria for AN, aged between 14 and 35 years, female	Pregnancy	DSM-5	NS	NS	NS	HRV (5 min)	Low
	HC	32F	NS	23.6 ± 5.3			No disordered eating, aged between 14 and 35 years, female							
Gross et al. (1979)	AN (T1)	15F	22 ± 2	NS	NS	NS	Met criteria or AN	NS	Halmi's criteria (Halmi et al., 1977)	Inpatient	NS	NS	MHPG (urinary), NE (plasma), Orthostatic response	Moderate
	AN-WR (T2)	13F	23 ± 2	NS	NS	NS	After significant weight gain							
	HC	39F	23 ± 1	NS			Age- and sex-matched AN group, normal BW	Medical or psychiatric illnesses						
Ishizawa et al. (2008)	AN	35F	22.9 ± 5.9	14.4 ± 2.0	Mean (range): 20 (4–192) months	19AN-R/16AN-BP	Met criteria for AN, aged between 16 and 35 years	Physical comorbidities aside from AN, medication that influences ANS function (SSRIs or tranquilizers)	DSM-IV	26 outpatient/9 inpatient	None	AN: 2 MDD, 1 dysthymic disorder	BPV, BRS, HRV (10 min)	Low
	HC	37F	24.3 ± 3.2	20.6 ± 1.4			Age- and sex-matched to AN group, BMI between 18.5 and 24.9	Any past or current physical or psychiatric disease and treatment with any drugs						
Kaye et al. (1990)	AN (T1)	NS	NS	NS	NS	NS	Met criteria for AN	NS	DSM-III	NS	NS	NS	NE (plasma)	High
	AN-WR (T2)	NS	NS	NS	NS	NS	Previously met criteria for AN	NS		NS	NS	NS		
	HC	11F	NS	NS			NS	NS						
Kaye et al. (1985)	AN-WR	11F	22.5 ± 3.3	NS	24 ± 19.8 months	AN-R/7 AN-BP	Previously met criteria for AN, long-term WR, stable BW for 6 months	Weight below 85% of IBW, medication use	DSM-III	Outpatient	Excluded	NS	MHPG (plasma and CSF), NE (plasma and CSF), Orthostatic response	Moderate
	HC	8F	25.6 ± 2.8	NS			Sex-matched to AN group, stable BW for 6 months	Medication use						
Kollai et al. (1994)	AN	11F	13.6 ± 1.8	NS	NS	NS	Met criteria for AN, <80% IBW for 6 months, medication free for 6 weeks	NS	DSM-III	Inpatient	None	AN: none met criteria for MDD	BRS (Valsalva Maneuver, Neck chamber technique, Atropine sulfate injection), HRV (5 min)	Moderate
	HC	11F	14.2 ± 1.9	NS			Height, age and sex-matched to AN group, no current or past psychopathology, normal menstrual cycles.	NS						
Koschke et al. (2010)	AN	20F	Mean (range): 24 (18–43)	Mean (range): 15.7 (13.2–18.5)	Mean (range): 70 (6–228) months	NS	Met DSM-IV criteria for AN, free from medical/psychiatric disease	History of peripheral neuropathies, cardiac arrhythmia, substance abuse	DSM-IV	Inpatient	None	None	HRV (30 min)	Low
	HC	20F	Mean (range): 25 (22–40)	Mean (range): 21.9 (17.9–24.8)			Age, physical activity- and sex-matched to AN group, free from medical/psychiatric disease							
Kreipe et al. (1994)	AN (T1)	8F	18.6 ± 3.9	NS	NS	All AN-R	Met criteria for AN	NS	DSM-III	Inpatient	NS	NS	HRV (Supine for 256 s, Orthostatic for 15 min), Orthostatic response	Moderate
	AN-WR (T2)	4F	NS	NS	NS	All AN-R	Previously met criteria AN, 2 weeks after inpatient treatment with variable amount of weight gain							
	HC	8F	20.9 ± 5.7	NS			Age- and sex-matched to AN group, normal diet and activity level							
Lachish et al. (2009)	AN (T1)	24F	15.9 ± 0.5	15.5 ± 0.3	2.5 ± 2.8 years	All AN-R	Met criteria for AN, stable medical condition	Lifetime or current diagnosis of SCX, BPD, SUD, organic brain disorder, CV disorder, medical disorder affecting food consumption/weight, taking any medication	DSM-IV	Inpatient	AN, HC, AN-WR (T3): None; AN-WR (T2): 8 taking SSRIs	AN: 1 OCD; 2 Social phobia; 7 MDD/dysthymia/ anxiety disorder	HRV (length NS)	Low
	AN-WR (T2)	12F	NS	19.5 ± 0.4	2 weeks	All AN-R	Previously met criteria for AN, at discharge when achieved desired BW for 2 weeks							
	AN-WR (T3)	6F	NS	21.5 ± 1.1	24–36 months	All AN-R	Previously met criteria for AN, 24–36 months WR							
	HC	19F	15.6 ± 0.5	21.0 ± 0.3			Age- and sex-matched to AN group, no lifetime or current medical/psychiatric disorder, weight within 85–115% of IBW since puberty, regular menstrual cycles	NS						
Lechin et al. (2010)	AN	22F	22.0 ± 6.4	NS	NS	10 AN-R, 12 AN-BP	Met criteria for AN, no other physical illness	Pregnancy, lactation, smoking and alcohol abuse	DSM-IV	NS	AN and HC: no medication for 15 days prior to study	NS	NE (plasma), Orthostatic response	Low
	HC	All F	NS	NS			Age-, sex-, and race-matched to AN group, no other physical illness							
Léonard et al. (1998)	AN	14F	23.9 ± 8.3	16.2 ± 3.1	5.9 ± 4.2 years	NS	Met criteria for AN	NS	DSM-IV	Inpatient	NS	NS	SCL	Moderate
	HC	18F	29.3 ± 9.7	21.1 ± 2.7			Age- and sex-matched to AN group							
Lesem et al. (1989)	AN (T1)	11F	25.2 ± 5.3	NS	NS	NS	Met criteria for AN, admitted to clinical unit	NS	DSM-III	Inpatient	AN and HC: no psychotropic medications for 4 weeks prior to study	NS	NE (plasma), Orthostatic response	Moderate
	AN-WR (T2)	11F	3 weeks after initial assessment	NS	NS	NS	Previously met criteria for AN, after 3 weeks of treatment (no significant change in BW)							
	HC	9F	27.6 ± 3.4	NS			Sex-matched to AN group, normal BW, no history or current psychiatric illness							
Lonigro et al. (2019)	AN	13F	15.0 ± 0.9	16.3 ± 1.5	NS	All AN-R	Met criteria for AN-R, female, aged between 13 and 17 years, absence of severe physical condition, no medication that may impact on ANS function	Diagnosed with intellectual disabilities, developmental disorders, SCX, or associated neurological conditions	DSM-5	Day patient	AN: no medication that may impact on autonomic functioning	AN: 2 MDD, 1 GAD	HRV (5 min)	Low
	HC	12F	15.3 ± 1.4	20.1 ± 1.2			Sex-matched to AN group, no lifetime history of ED or psychiatric disorder	NS						
Luck et al. (1983)	AN	18F	Mean: 17.5	NS	NS	NS	Met criteria for AN, normal plasma sodium levels, no evidence of de- or over-hydration	NS	Russell's criteria (Russell, 1970)	Inpatient and outpatient	No medication 2 weeks prior to study	NS	NE (plasma)	Moderate
	HC	20F	Mean: 18.0	NS			Age- and sex-matched to AN group, no recent weight loss, normal plasma sodium levels, no clinical evidence of de- or over-hydration							
Lutz et al. (2019)	AN	19F	25.2 ± 5.3	15.79 ± 1.67	9.7 ± 6.7 years	10 AN-R, 9 AN-BP	Met criteria for AN; >18 years age	Past or current psychotic disorders, current substance use, current PTSD	DSM-5	Inpatient	AN: 6 taking SSRIs	6 had between 1 and 3 comorbid diagnoses of MDD, OCD, agoraphobia or BPD	HRV (5 min)	Low
	HC	19F	24.6 ± 3.9	22.3 ± 3.2			Age- and sex-matched to AN group; >18 years age	Past or current ED or mental disorders, current substance use						
Mazurak et al. (2011b)	AN	21F	23.8 ± 6.9	16.1 ± 1.5	NS	NS	Met criteria for AN	Taking medication, pregnant or lactating, history of alcohol or drug abuse or failed to follow instructions during testing	ICD-10	Inpatient	None	NS	HRV (3 min)	Low
	HC	21F	25.0 ± 8.1	20.1 ± 1.5			Age- and sex-matched to AN group, no history of AN, IBS or chronic somatic or psychiatric disorder							
Melanson et al. (2004)	AN	6F	29 ± 3	NS	Mean (range): 19 (2–72) months	NS	Met criteria for AN	NS	DSM-IV	Outpatient	NS	NS	HRV (5min; 24 h)	Moderate
	HC	10F	24 ± 3	NS			Sex-matched to AN group, no history of congenital or acquired heart disease or hypertension	NS						
Murialdo et al. (2007)	AN	34F	Mean (CI): 24.2 (21.4–27.1)	Mean (CI): 15.7 (15.1–16.4)	Mean (CI): 55.4 (38.6–72.1) months	NS	Met criteria for AN	NS	DSM-IV	Inpatient	NS	NS	HRV (330 s), Orthostatic response	Moderate
	HC	30F	Mean (CI): 25.9 (23.3–28.5)	Mean (CI): 22.9 (22.3–23.6)			Sex-matched to AN group, normal menstrual cycle and BMI	NS						
Nakai et al. (2015)	AN	14F	27.9 ± 9.4	13.2 ± 1.9	121.9 ± 105.3 months	All AN-R	Met criteria for AN-R subtype	Anxiety disorders, emotional disorders	DSM-IV	Inpatient	None	NS	HRV (5 min)	Low
	HC	22F	23.2 ± 4.2	18.5 ± 1.7			Age- and sex-matched to AN group, no current Axis I disorder, no history of EDs or CV diseases	NS						
Nedvidkova et al. (2004)	AN	5F	23.0 ± 1.3	14.7 ± 0.7	NS	NS	Met criteria for AN, non-smokers, no allergies, medication free for 1 month prior to the study	NS	DSM-IV	Inpatient	None	NS	NE (plasma and adipose tissue)	Low
	HC	6F	22.3 ± 1.0	22.2 ± 0.3			Age- and sex-matched to AN group, non-smokers, no allergies, medication free for 1 month prior to the study, normal ECG	History of obesity, malnutrition, endocrine or CV disease, EDs, psychiatric disorders						
Palomba et al. (2017)	AN	13 (sex NS)	22.31 ± 7.74	15.69 ± 1.64	7.46 ± 7.37 years	11 AN-R, 2 AN-BP	Met criteria for AN	Medical pathologies, pregnancy or nursing, SUD, medication use that affects ANS function	DSM-IV	Outpatient	Excluded	Excluded	SCL	Low
	HC	13 (sex NS)	23.92 ± 3.38	20.31 ± 1.66			Age-matched to AN group, ED pathology scores 1 SD below normal							
Petretta et al. (1997)	AN	13F	20 ± 2	15.7 ± 2.4	NS	All AN-R	Met criteria for AN-R subtype, BMI <19, stable BW for 3 months prior to study	AN-BP subtype	DSM-IV	Outpatient	NS	AN: 5 mood disturbance with depressive features, 2 of these had MDD diagnosis	HRV (24 h)	Low
	HC-T	10F	22 ± 3	16.6 ± 1.1			BMI <20, normal scores on the food questionnaire	Met criteria for AN						
	HC-NW	10F	21 ± 3	23.4 ± 2.4			BMI between 20 and 27, normal scores on the food questionnaire							
Pirke et al. (1992)	AN	NS	22.9 ± 9.8	15.1 ± 1.5	3.6 ± 4.6 years	6AN-R, 1AN-BP	Met criteria for AN	NS	DSM-III	Inpatient	AN, AN-WR: NS; HC: none	NS	NE (plasma)	Moderate
	AN-WR	NS	25.4 ± 3.7	20.0 ± 4.5	4.0 ± 2.0 years	8AN-R, 2AN-BP	NS	NS						
	HC	12F	23.5 ± 1.9	21.5 ± 1.4			Age-matched to AN group, female	Currently dieting, medication use (except contraceptive pill), alcohol or drug use, heavy exercise						
Platisa et al. (2006)	AN-C	9F	21.0 ± 9.0	14.4 ± 0.6	36 ± 9 months	NS	Met criteria for AN; divided into two groups according to duration of AN	NS	DSM-IV	Inpatient	None	NS	HRV (24 h)	Low
	AN-A	8F	20.4 ± 0.8	15.7 ± 0.6	12 ± 15 months	NS		NS						
	HC	8F	20.4 ± 0.8	20.6 ± 0.4			NS	NS						
Rechlin et al. (1998)	AN	16F, 2M	Mean: 23.8 years	NS	NS	NS	Met criteria for AN, <75% IBW	Psychotropic medication use in 3 months prior to study, diabetes mellitus, CV or neurological diseases, diagnosis of MDD or panic disorder	DSM-III-R	Inpatient	Excluded	AN, AN-PWR, AN-WR: MDD and panic disorder excluded	HRV (5 min), Valsalva Maneuver	Moderate
	AN-PWR	17F, 1M	Mean: 22.0 years	NS	NS	NS	Previously met criteria for AN, between 76 and 90% IBW							
	AN-WR	11F, 1M	Mean: 23.5 years	NS	NS	NS	Previously met criteria for AN, >90% IBW							
	HC	14F, 2M	Mean: 23.4 years	NS			Age- and sex-matched to AN group	NS						
Riederer et al. (1982)	AN (T1)	14F, 2M	15.3 ± 0.5	NS	NS	NS	Met criteria for AN	NS	Feighner's criteria (Feighner et al., 1972)	Inpatient	1 taking benzodiazepines	NS	MHPG (urine)	Moderate
	AN-WR (T2)	NS	NS				Previously met criteria for AN, post-treatment							
	HC	11F, 9M	17.6 ± 2.8	NS			Normal BW	NS						
Roche et al. (2004)	AN	23F, 2M	19.0 ± 3.0	15.2 ± 2.1	NS	NS	Met criteria for AN	Diabetes mellitus, systemic hypertension, atrial fibrillation, implanted pacemaker	DSM-IV	Inpatient	NS	NS	HRV (24 h)	Moderate
	HC	23F, 2M	19.0 ± 2.0	22.5 ± 1.9			Age- and sex-matched to AN group	NS						
Rommel et al. (2015)	AN	NS	Median (1st-3rd Q): 19.5 (17.3–21)	Median (1st-3rd Q): 15.2 (14.5–15.4)	Median (1st-3rd Q): 3.5 (2–5) years	All AN-R	Met DSM-IV criteria for AN; average BMI of 15	AN-BP, high anxiety, neurological disorders, PTSD, SUD, intellectual deficits, missing data	DSM-IV	Inpatient	NS	NS	HRV (2 min)	Low
	HC	24F	Median (1st-3rd Q): 19 (19-21)	Median (1st-3rd Q): 21.0 (19.6-22.3)			Age- and education-matched to AN group							
Russell et al. (2008)	AN	17F	23.6 ± 10.6	16.2 ± 2.2	NS	NS	Met criteria for AN	NS	DSM-IV and ICD-10	Inpatient	Patients were started on antidepressants and antipsychotic agents depending on clinical need	NS	HRV (20 min)	Moderate
	HC	35F	22.9 ± 5.0	21.3 ± 3.0			Age- and sex-matched to AN group; stable BW	NS						
Takimoto et al. (2014)	AN	21F	23.9 ± 6.0	14.9 ± 1.9	50 (7–192) months	13 AN-R, 8 AN-BP	Met criteria for AN	Mental and physical diseases not related to AN, medication that might influence ANS function, patients with OH or POTS	DSM-IV	10 outpatient/11 inpatients	Excluded	Excluded	BPV, BRS, HRV, Orthostatic response	Low
	HC	30F	22.5 ± 3.3	20.7 ± 1.6			Age- and sex-matched to AN group	NS						
Tonhajzerova et al. (2020)	AN	20F	14.6 ± 2.1	16.4 ± 2.6	8.6 ± 2.8 months	All AN-R	Met criteria for AN; AN-R subtype	AN-BP (AN group), smoking, CV, respiratory, endocrine, neurological, metabolic, or infectious diseases or mental disorders (excluding AN), medication or dietary supplementation which could affect CV or ANS function	DSM-5	Inpatient	Excluded	Excluded	BPV, BRS, HRV	Low
	HC	20F	16.1 ± 1.0	21.6 ± 2.9			Age- and sex-matched to AN group							
Van Binsbergen et al. (1991)	AN	10F	Mean: 24.5 years	Mean: 14.3	NS	NS	Met criteria for AN; aged between 18 and 35; weight <75% IBW; no medication use; amenorrhea for 6 months; acrocyanosis; lanugo	Substance use, psychotropic medication usage, professional athlete	DSM-III	Outpatient	Excluded	NS	MHPG (24-h urine excretion), NE (plasma; 24-h urine excretion), Orthostatic response	Low
	HC-T	10F	Mean: 26.4years	Mean: 18.1			Sex-matched to AN group, regular ovulatory cycles, 80–90% IBW	Acrocyanosis, lanugo, distorted attitudes toward eating, food, or weight, extreme fear of becoming obese, professional athlete						
	HC-NW	10F	Mean: 25.1	Mean: 20.7			Sex-matched to AN group, regular ovulatory cycles, 90–120% IBW							
Vigo et al. (2008)	AN	14F	26.6 ± 8.0	17.7 ± 2.2	Range of 0.5–15 years	All AN-R	Met criteria for AN	Abnormal cardiac rhythm, anticholinergic medication use in week prior to the study, psychotic symptoms. AN: history of binging	DSM-IV	Outpatient	Excluded	Excluded	HRV (10 min)	Low
	HC	19F	26.2 ± 1.8	20.2 ± 1.1			Age- and sex-matched to ED group							
Wu et al. (2004)	AN	14F	18.5 ± 5.0	13.2 ± 2.0	NS	NS	Met criteria for AN	Autonomic disorders, diabetes mellitus, CV diseases or arrhythmias	DSM-IV	Inpatient	NS	NS	HRV (5 min)	Low
	HC	12F	19.5 ± 1.2	21.2 ± 1.4			No family history of hypertension or CV disease							

*AN, anorexia nervosa; AN-A, acute AN; AN-BP, anorexia nervosa, binge-purge subtype; AN-C, chronic AN; AN-PWR, previous diagnosis of anorexia nervosa, partial weight-restoration; AN-R, anorexia nervosa, restricting subtype; ANS, autonomic nervous system; AN-WR, previous diagnosis of anorexia nervosa, weight-restored; BP, blood pressure; BPD, bipolar disorder; BPV, blood pressure variability; BRS, baroreflex sensitivity; BW, body weight; CI, confidence interval; CV, cardiovascular; DSM-5, Diagnostic and Statistical Manual of Mental Disorders, 5th edition; DSM-III, Diagnostic and Statistical Manual of Mental Disorders, 3rd edition; DSM-IV, Diagnostic and Statistical Manual of Mental Disorders, 4th edition; ECG, electrocardiogram; ED, eating disorder; F, female; GAD, Generalized anxiety disorder; HC, healthy controls; HC-NW, Normal weight healthy controls; HC-T, Constitutionally thin healthy controls; HRV, heartrate variability; IBS, Irritable bowel syndrome; IBW, ideal body weight; ICD-10, International Classification of Diseases, Tenth Revision; IQ, Intelligence Quotient; M, male; MDD, Major depressive disorder; MHPG, 3-methyl-4-hydroxyphenylglycol; NE, noradrenaline; NS, not specified; OCD, obsessive compulsive disorder; ODD, oppositional defiant disorder; OH, orthostatic hypotension; PLR, pupillometry response; PTSD, post-traumatic stress disorder; SCL, skin conductance level; SCX, schizophrenia spectrum disorder; SD, standard deviation; SSRI, Selective Serotonin Reuptake Inhibitor; SUD, substance use disorder; T1, time 1; T2, time 2; T3, time 3; Q, quartile*.

### Study Quality Assessment

The NOS scores of the included studies ranged from 3 to 10. Among the 46 included studies, two were at high risk of bias (4.3%), 17 were at moderate risk (37.0%), and 27 were at low risk (58.7%) (see Table 2 for classification or detailed assessment in Appendix 4 in Supplementary Material). The HRV quality summary score is listed in Table 3 (see Appendix 5 in Supplementary Material for a detailed assessment).

**Table 3:** Heart-rate variability, as compared to controls.

**References**	**HRV duration**	**Group**	**SDNN/RR**	**RMSSD**	**NN50/pNN50**	**TP**	**LF**	**HF**	**LF/HF**	**α**	**Outcome**	**Quality (score between 0 and 22)**
Bar et al. (2006)	5 min	AN		↑					NS		Increased pain thresholds are associated with increased parasympathetic tone in AN but not AN-WR	12
	5 min	AN-WR		NS					NS			
Billeci et al. (2015)	15 min	AN	↑	↑			↓	↑	↓		Prevalence of parasympathetic over sympathetic activity in AN	14
Billeci et al. (2019)	5 min	AN	↑	↑			↓	↑	↓		Patients with AN demonstrated an altered autonomic response to light exercise (indicated SNS activation and PNS withdrawal)	19
Bomba et al. (2014)	24-h	AN	↑	↑	NS		NS	↑	↓		Patients with AN demonstrated increased PNS at rest, which were mirrored (to a lesser magnitude) by patients with functional hypothalamic amenorrhea	19
Casu et al. (2002)	5 min each: supine and orthostatic	AN	Supine NS; Orthostatic ↓				NR	NR	NR		Patients with AN demonstrated abnormally persistent parasympathetic HRV modulation during an orthostatic challenge	7
Galetta et al. (2003)	24-h	AN	↑	↑	↑		NS	↑	↓		Patients with AN have a preserved sympathetic and increased parasympathetic HRV response to an orthostatic challenge, which may be compensatory mechanisms to starvation	14
Green et al. (2020)	5 min	AN						NS	NS		There was no difference in cardiac autonomic balance between individuals with AN and HCs	13
Ishizawa et al. (2008)	10 min	AN				↑	NS	↑	↓	↓	HRV in patients with AN demonstrated decreased SNS, increased PNS and increased complexity of inter-beat intervals, which may be a protective cardiovascular mechanism	19
Kollai et al. (1994)	5 min	AN	↑								Patients with AN demonstrated high resting vagal activity	8
Koschke et al. (2010)	30 min	AN		↑					↓		AN patients demonstrated resting vagal predominance which remained after controlling for BMI	19
Kreipe et al. (1994)	Supine: 256 s: Orthostatic: 15 min	AN (T1)					Supine ↓; Orthostatic NS	NS	Supine ↓; Orthostatic NS		AN patients demonstrated persistent PNS modulation in response to an orthostatic challenge and decreased resting SNS activity. This trended toward control values after 2 weeks of inpatient treatment	10
		AN-WR (T2)					NS	NS	NS			
Lachish et al. (2009)	NR	AN (T1)	↓			↓	↓	↑	↓		Individuals with AN demonstrated cardiovascular vagal hyperactivity, which persisted after short- and long-term weight restoration	17
	NR	AN-WR (T2)	↓			↓	↓	↑	↓			
	NR	AN-WR (T3)	↓			↓	↓	↑	↓			
Lonigro et al. (2019)	5 min	AN						NS			Patients with AN demonstrated delayed recovery and stronger PNS activity in response to emotional attachment test, which may reflect altered emotion regulation	13
Lutz et al. (2019)	5 min	AN						NS			Patients with AN demonstrated no significant difference in sympathetic cardiac modulation to HCs	15
Mazurak et al. (2011b)	3 min each: supine, orthostatic, recovery	AN						Supine, recovery NS; Orthostatic ↑			Patients with AN longer inter-beat intervals and weaker vagal withdrawal during an orthostatic test (independent from BMI)	18
Melanson et al. (2004)	5 min; 24 h	AN		Short-term NS; 24-h, daytime: ↓	Short-term NS; 24-h, daytime, night-time: ↓		Short-term NS; 24-h, daytime, night-time: ↓	Short-term NS; 24-h, daytime, night-time: ↓	NS		Patients in various stages of recovery and refeeding demonstrated decreased resting and ambulatory measures of HRV with decreased parasympathetic activity	12
Murialdo et al. (2007)	5.5 min each: supine, orthostatic	AN					Supine NS; Orthostatic ↓	NS			Patients with AN demonstrated reduced SNS response to an orthostatic challenge. Disease duration and BMI were not correlated with HRV parameters	10
Nakai et al. (2015)	5 min	AN					NS	NS	NS		Illness duration was negatively correlated with increased parasympathetic tone (HF) and positively correlated with lower vagal tone/high sympathetic tone (LF/HF ratio). This may indicate an initial adaptive response in AN with increased cardiac risk over a longer illness duration	21
Petretta et al. (1997)	24 h	AN	24-h, daytime, night-time ↑	24-h, daytime, night-time ↑	24-h, daytime, night-time ↑	24-h, daytime ↑; night-time NS	24-h, night-time ↑; daytime NS	24-h, daytime, night-time ↑			Patients demonstrated longer inter-beat intervals and increased parasympathetic activity over 24-h compared to thin and normal weight controls	19
Platisa et al. (2006)	24 h	AN-C	NS				↓	↓		NS	Difference in HRV measures for acute and chronic AN; increased HRV in acute AN and decreased HRV in chronic AN. This may indicate the compensatory increased PNS tone in acute AN is attenuated over illness duration	16
		AN-A	NS				↑	↑		**↓**		
Rechlin et al. (1998)	5 min each: supine and orthostatic	AN					↓	↓			Patients with AN demonstrated decreased sympathetic activity at rest and in response to an orthostatic challenge which trended to be reversed with weight restoration. Resting and orthostatic SNS activity were positively correlated with body weight.	15
		AN-PWR					↓	NS				
		AN-WR					↓	NS				
Roche et al. (2004)	24 h	AN	↑	↑	NS	NS	↓	↑			24-h HRV assessment demonstrated enhanced parasympathetic activity and withdrawal of sympathetic control	13
Rommel et al. (2015)	2 min	AN						↓			In response to an emotional induction test, patients with AN displayed increased time required for parasympathetic activity to return to baseline, which may be a physiological disturbance due to emotion regulation deficits	14
Russell et al. (2008)	20 min	AN	NS	NS	↓		NS	NS	NS	NS	Patients with AN demonstrated decreased pNN50 compared to HCs but did not differ in any other HRV parameters. HRV parameters in AN group differed from individuals with other EDs	9
Tonhajzerova et al. (2020)	5 min	AN						NS			There were no significant differences in cardiac-linked vagal modulation indexed by HRV HF in patients with anorexia nervosa, compared to controls	21
Vigo et al. (2008)	10 min	AN	NS	NS			**↓**	NS		**↓**	Patients with AN demonstrated decreased HRV fractal scaling exponent and LF than controls. This pattern is similar to patients after myocardial infarction and may represent increased randomness of HR.	17
Wu et al. (2004)	5 min	AN				NS	↑	↑	↓		Patients with AN demonstrated decreased SNS and enhanced PNS HRV activity. SNS (LF) was negatively correlated with anxiety and illness duration whereas PNS (HF) was positively associated with anxiety and illness duration	13

*AN, anorexia nervosa; AN-A, acute anorexia nervosa; AN-C, chronic anorexia nervosa; AN-PWR, previous diagnosis of anorexia nervosa, partially weight-restored; AN-WR, previous diagnosis of anorexia nervosa, weight-restored; BMI, body mass index; ED, eating disorder; HC, healthy controls; HF, High frequency power; HRV, heartrate variability; LF/HF, Low/high frequency power; LF, Low frequency power; NN50, Normal-to-normal intervals >50 ms (% of); NR, not reported; NS, not significant; PNS, parasympathetic nervous system; RMSSD, Root Mean Square of Successive Differences; SDNN, Standard deviation of normal-to-normal intervals; SNS, sympathetic nervous system; TP, Total power; T1, time 1; T2, time 3; T3, time 3; α, Short-term fractal scaling exponent*.

### Heartrate Variability

Resting HRV was an outcome measure in 27 articles (Kollai et al., 1994; Kreipe et al., 1994; Petretta et al., 1997; Rechlin et al., 1998; Casu et al., 2002; Galetta et al., 2003; Melanson et al., 2004; Roche et al., 2004; Wu et al., 2004; Bar et al., 2006; Platisa et al., 2006; Murialdo et al., 2007; Ishizawa et al., 2008; Russell et al., 2008; Vigo et al., 2008; Lachish et al., 2009; Koschke et al., 2010; Mazurak et al., 2011b; Bomba et al., 2014; Billeci et al., 2015, 2019; Nakai et al., 2015; Rommel et al., 2015; Lonigro et al., 2019; Lutz et al., 2019; Green et al., 2020; Tonhajzerova et al., 2020) (see Table 3). Studies used various durations of HRV assessment; 21 reported HRV outcomes from short-term recordings (Kollai et al., 1994; Kreipe et al., 1994; Rechlin et al., 1998; Casu et al., 2002; Wu et al., 2004; Bar et al., 2006; Murialdo et al., 2007; Ishizawa et al., 2008; Russell et al., 2008; Vigo et al., 2008; Lachish et al., 2009; Koschke et al., 2010; Mazurak et al., 2011b; Billeci et al., 2015, 2019; Nakai et al., 2015; Rommel et al., 2015; Lonigro et al., 2019; Lutz et al., 2019; Green et al., 2020; Tonhajzerova et al., 2020), five used ambulatory HRV recordings taken over 24 h (Petretta et al., 1997; Galetta et al., 2003; Roche et al., 2004; Platisa et al., 2006; Bomba et al., 2014) and one study reported both (Melanson et al., 2004). Regarding the HRV measures reported, 15 of the included studies reported on time domain HRV (Kollai et al., 1994; Petretta et al., 1997; Casu et al., 2002; Galetta et al., 2003; Melanson et al., 2004; Roche et al., 2004; Bar et al., 2006; Platisa et al., 2006; Russell et al., 2008; Vigo et al., 2008; Lachish et al., 2009; Koschke et al., 2010; Bomba et al., 2014; Billeci et al., 2015, 2019), 16 reported low frequency (LF) (Kreipe et al., 1994; Petretta et al., 1997; Rechlin et al., 1998; Casu et al., 2002; Galetta et al., 2003; Melanson et al., 2004; Roche et al., 2004; Wu et al., 2004; Platisa et al., 2006; Murialdo et al., 2007; Ishizawa et al., 2008; Lachish et al., 2009; Bomba et al., 2014; Billeci et al., 2015, 2019; Nakai et al., 2015), 22 reported high frequency (HF) (Kreipe et al., 1994; Petretta et al., 1997; Rechlin et al., 1998; Casu et al., 2002; Galetta et al., 2003; Melanson et al., 2004; Roche et al., 2004; Wu et al., 2004; Platisa et al., 2006; Murialdo et al., 2007; Ishizawa et al., 2008; Lachish et al., 2009; Mazurak et al., 2011b; Bomba et al., 2014; Billeci et al., 2015, 2019; Nakai et al., 2015; Rommel et al., 2015; Lonigro et al., 2019; Lutz et al., 2019; Green et al., 2020; Tonhajzerova et al., 2020) and 14 studies reported on calculated ratios of LF to HF (LF/HF) (Kreipe et al., 1994; Casu et al., 2002; Galetta et al., 2003; Melanson et al., 2004; Wu et al., 2004; Bar et al., 2006; Ishizawa et al., 2008; Lachish et al., 2009; Koschke et al., 2010; Bomba et al., 2014; Billeci et al., 2015, 2019; Nakai et al., 2015; Green et al., 2020). Four studies used detrended fluctuation analysis (DFA) to calculate the scaling exponent (α) of HRV (Platisa et al., 2006; Ishizawa et al., 2008; Russell et al., 2008; Vigo et al., 2008).

#### (i) Current AN

Basal time domain HRV in individuals with a current diagnosis of AN was reported in 15 studies. Of the ten studies that assessed short-term time domain HRV, individuals with AN had increased HRV in five studies (Kollai et al., 1994; Bar et al., 2006; Koschke et al., 2010; Billeci et al., 2015, 2019), decreased in two studies (Russell et al., 2008; Lachish et al., 2009) and unchanged in three studies (Casu et al., 2002; Melanson et al., 2004; Vigo et al., 2008), as compared to controls. Of the six studies that assessed time domain HRV using ambulatory recordings, four reported increased HRV (Petretta et al., 1997; Galetta et al., 2003; Roche et al., 2004; Bomba et al., 2014), one reported decreased HRV (Melanson et al., 2004) and one reported unchanged HRV (Platisa et al., 2006). Two studies demonstrated increased time domain HRV, as compared to lean controls (Petretta et al., 1997; Galetta et al., 2003).

Frequency domain HRV was assessed in 20 short-term recordings and 6 ambulatory recordings. Of the 16 studies that reported resting LF, seven reported decreased LF (Kreipe et al., 1994; Rechlin et al., 1998; Roche et al., 2004; Vigo et al., 2008; Lachish et al., 2009; Billeci et al., 2015, 2019), two reported increased LF (Petretta et al., 1997; Wu et al., 2004) and six reported unchanged LF (Galetta et al., 2003; Murialdo et al., 2007; Ishizawa et al., 2008; Russell et al., 2008; Bomba et al., 2014; Nakai et al., 2015), compared to controls. One study reported decreased LF HRV in acute AN (average illness duration of 12 months) and increased LF in chronic AN (average illness duration of 36 months) (Platisa et al., 2006). Of the 22 studies that reported resting HF in individuals with a current diagnosis of AN, nine reported increased HF (Petretta et al., 1997; Galetta et al., 2003; Roche et al., 2004; Wu et al., 2004; Ishizawa et al., 2008; Lachish et al., 2009; Bomba et al., 2014; Billeci et al., 2015, 2019), two reported decreased HF (Rechlin et al., 1998; Roche et al., 2004) and ten reported unchanged HF (Kreipe et al., 1994; Murialdo et al., 2007; Russell et al., 2008; Vigo et al., 2008; Mazurak et al., 2011b; Nakai et al., 2015; Lonigro et al., 2019; Lutz et al., 2019; Green et al., 2020; Tonhajzerova et al., 2020), as compared to controls. Platisa et al. (2006) found increased HF in acute AN and decreased HF in chronic AN, while Melanson et al. (2004) assessed both short-term and ambulatory HRV, reporting no difference between groups in short-term recording of LF or HF, yet ambulatory results demonstrated both decreased LF and HF in individuals with AN, compared to controls. Of the 14 studies that reported a calculated LF/HF ratio, nine reported decreased LF/HF ratio (Kreipe et al., 1994; Galetta et al., 2003; Wu et al., 2004; Ishizawa et al., 2008; Lachish et al., 2009; Koschke et al., 2010; Bomba et al., 2014; Billeci et al., 2015, 2019) and five reported unchanged LF/HF ratios (Melanson et al., 2004; Bar et al., 2006; Russell et al., 2008; Nakai et al., 2015; Green et al., 2020), as compared to controls.

Of the four studies that reported non-linear assessments of HRV and reported the scaling exponent (α), two found decreased α (Ishizawa et al., 2008; Vigo et al., 2008) and one reported no difference in α compared to controls (Russell et al., 2008). Platisa et al. (2006) again highlighted differences according to duration of AN, reporting decreased α in those with a shorter illness duration and no difference to controls in those with an extended illness duration.

Overall, three studies indicated differences in HRV modulation according to duration of illness. A shorter illness duration was demonstrated by increased parasympathetic modulation which was attenuated over time in two studies (Platisa et al., 2006; Nakai et al., 2015). However, Wu et al. (2004) found a negative correlation between enhanced SNS activity and illness duration and a positive correlation between PNS activity and illness duration.

#### (ii) Weight-Restored AN

Four studies reported on HRV in individuals with a previous diagnosis of AN who were in varying stages of weight restoration. Two reported time domain HRV; one reported decreased HRV (no change from the current AN group) as compared to controls (Lachish et al., 2009) and the other reported no difference between AN-WR and controls (Bar et al., 2006). Three reported LF HRV in AN-WR; two reported maintenance of decreased LF (Rechlin et al., 1998; Lachish et al., 2009) and one reported no difference in LF (Kreipe et al., 1994) between AN-WR and controls. The same three studies also recorded HF in AN-WR; one reported maintenance of high HF in AN-WR (Lachish et al., 2009) and two reported no difference in HF (Kreipe et al., 1994; Rechlin et al., 1998) between AN-WR and controls. Three studies calculated the LF/HF ratio; one reported sustained low LF/HF after weight restoration (Lachish et al., 2009) and two reported no difference in LF/HF between AN-WR and controls (Kreipe et al., 1994; Bar et al., 2006).

### Orthostatic Response, Blood Pressure Variability, and Baroreflex Sensitivity

Eleven studies reported the response to an orthostatic challenge, blood pressure variability (BPV), or baroreflex sensitivity (BRS) as an outcome measure (Gross et al., 1979; Lesem et al., 1989; Van Binsbergen et al., 1991; Kollai et al., 1994; Kreipe et al., 1994; Casu et al., 2002; Murialdo et al., 2007; Ishizawa et al., 2008; Lechin et al., 2010; Takimoto et al., 2014; Tonhajzerova et al., 2020) (see Table 4).

**Table 4:** Orthostatic response, blood pressure variability and baroreflex sensitivity, as compared to controls.

**References**	**Variables assessed**	**Group**	**Orthostatic SBP**	**Orthostatic DBP**	**BPV**	**BRS**	**NE**	**Outcome**
Casu et al. (2002)	Orthostatic BP	AN	↓	↓				Patients with AN demonstrated discorded sympathovagal balance during an orthostatic challenge, with a trend toward a high degree of vagal tone
Gross et al. (1979)	Orthostatic BP, NE	AN	↓	NS			↓	AN-WR patients demonstrated higher BP than acute AN patients, yet the levels were still below controls in response to an orthostatic challenge.
		AN-WR	↓	NS			NS	
Ishizawa et al. (2008)	BPV, BRS	AN			LF ↓	↑		Patients with AN demonstrated decreased LF variability of BPV, indicating decreased SNS responsiveness. Increased BRS is associated with increased PNS responsiveness.
Kollai et al. (1994)	BRS	AN				↑		Patients with AN demonstrated high resting vagal activity, which is partly explained by enhanced BRS and likely contributes to bradycardia
Kreipe et al. (1994)	Orthostatic BP	AN	↓					AN patients demonstrated abnormal autonomic control of cardiovascular activity in response to an orthostatic challenge, showing low sympathetic modulation compared to HCs. This trended toward control values after 2 weeks of inpatient treatment
Lechin et al. (2010)	Orthostatic BP	AN	NS	NS			↓	There were no significant variations in BP in individuals with AN or controls in response to orthostasis
Lesem et al. (1989)	Orthostatic BP, NE	AN (T1)	↓	↓			↑	Patients with AN demonstrated decreased BP in response to an orthostatic challenge, which trended toward controls after weight stabilization. NE levels were higher in individuals with AN during orthostasis, and trended toward normal levels following weight restoration
		AN-WR (T2)	↓	↓			↓	
Murialdo et al. (2007)	Orthostatic BP	AN	↓	↓				Patients with AN demonstrated reduced BP response to an orthostatic challenge, compared to HCs
Takimoto et al. (2014)	Orthostatic SBP, BPV, BRS	AN	NS		LF ↓	Phase shift ↑		Individuals with AN demonstrated altered autonomic changes in response to head-up tilting. Activation of the SNS was weak, whereas the PNS was strongly inhibited
Tonhajzerova et al. (2020)	BPV, BRS	AN			LF ↓	NS		At rest, individuals with AN demonstrated lower sympathetically mediated BPV, indicative of insufficient sympathetic cardiovascular control. BRS was significantly higher than participants with obesity but not controls.
Van Binsbergen et al. (1991)	Orthostatic NE response	AN					NS	The NE response to a postural change did not differ between individuals with AN and HCs

#### (i) Current AN

Six studies assessed BP response to an orthostatic challenge in individuals with a current diagnosis of AN; five reported decreased systolic BP (SBP) and/or diastolic BP (DBP) (Gross et al., 1979; Lesem et al., 1989; Kreipe et al., 1994; Casu et al., 2002; Murialdo et al., 2007) and one did not directly compare the response to controls (Lechin et al., 2010). Four studies investigated NE levels in response to an orthostatic challenge; two found a decreased response (Gross et al., 1979; Lechin et al., 2010), one an increased response (Lesem et al., 1989), and one found no difference (Van Binsbergen et al., 1991), as compared to controls.

Three studies reported increased BRS in individuals with a current diagnosis of AN (Kollai et al., 1994; Ishizawa et al., 2008; Takimoto et al., 2014) but Tonhajzerova et al. (2020) reported no difference in BRS to controls. All three studies that assessed BPV in individuals with AN reported decreased LF variability of BP (Ishizawa et al., 2008; Takimoto et al., 2014; Tonhajzerova et al., 2020).

#### (ii) Weight-Restored AN

Two studies assessed BP response to an orthostatic challenge in AN-WR groups, with both reporting maintenance of decreased BP response (Gross et al., 1979; Lesem et al., 1989). However, both reports of NE response to an orthostatic challenge were no different from controls (Gross et al., 1979), or trended toward control levels (Lesem et al., 1989) following weight restoration.

### Adrenergic Assessment

Fourteen studies reported basal NE or MHPG as an outcome measure (Gross et al., 1979; Riederer et al., 1982; De Rosa et al., 1983; Luck et al., 1983; Kaye et al., 1985, 1990; Lesem et al., 1989; Van Binsbergen et al., 1991; Pirke et al., 1992; Bartak et al., 2004; Nedvidkova et al., 2004; Dostalova et al., 2007; D'Andrea et al., 2008; Lechin et al., 2010) (see Table 5).

**Table 5:** Basal noradrenaline and MHPG levels, as compared to controls.

**References**	**Group**	**Plasma NE**	**Urinary NE**	**Adipose tissue NE**	**Plasma MHPG**	**Urinary MHPG**	**Outcome**
Bartak et al. (2004)	AN	NS		↑			Basal plasma NE levels were not different between AN and control groups, but controls demonstrated increased NE levels during exercise. The local SNS activity in abdominal adipose tissue was increased in AN patients
D'Andrea et al. (2008)	AN	↓					Individuals with AN and BN demonstrated decreased basal levels of urinary NE compared to controls. AN and BN differed in other measures of biochemical profile
De Rosa et al. (1983)	AN		↓				Decreased basal urinary NE was accompanied by multiple endocrine abnormalities in AN which are consistent with hypothalamic dysfunction
Dostalova et al. (2007)	AN	NS					No difference in basal or exercise-induced plasma NE levels between individuals with AN and controls
Gross et al. (1979)	AN	↓				↓	Plasma NE and urinary excretion of MHPG were lower in AN group than controls but increased to normal levels after weight restoration, suggesting that they were secondary to malnutrition and not etiological factors
	AN-WR	NS				NS	
Kaye et al. (1990)	AN (T1)	NS					No difference in basal NE levels between individuals with acute AN, during refeeding or after weight restoration, compared to controls
	AN-WR (T2)	NS					
Kaye et al. (1985)	AN-WR	↓			↓		Reduced noradrenergic activity was present in long-term weight restored individuals
Lechin et al. (2010)	AN	NR					Individuals with AN demonstrated predominance of circulating adrenaline over NE during resting, orthostasis and exercise
Lesem et al. (1989)	AN (time 1)	Supine NS; Orthostatic ↑					Patients with AN had elevated plasma NE levels at hospital admission, which gradually declined over 3 weeks of treatment
	AN-WR (time 2)	Supine NS; Orthostatic ↓					
Luck et al. (1983)	AN	↓					Resting plasma NE levels were reduced in patients with AN, which may be a consequence of SNS suppression in response to starvation
Nedvidkova et al. (2004)	AN	NS		↑			Patients with AN demonstrated increased basal adipose tissue NE levels, but no difference in plasma NE levels, demonstrating the existence of different SNS activity at whole body level and at adipose tissue level
Pirke et al. (1992)	AN	↓					Basal plasma NE levels were lower in AN and AN-WR individuals, compared to HCs. Patients with AN also demonstrated a lower plasma NE to a test meal. Plasma NE was negatively correlated with 'eating restraint', which may be the causal factor for NE suppression despite weight restoration
	AN-WR	↓					
Riederer et al. (1982)	AN (T1)					↓	Food intake and body composition influence urinary MHPG, with normalization after treatment
	AN-WR (T2)					NS	
Van Binsbergen et al. (1991)	AN	Supine ↑; Orthostatic NS	↓			NS	Patients with AN had higher plasma NE levels at rest but a normal NE plasma response to an orthostatic challenge. AN patients had lower urinary NE excretion levels than lean controls, which may reflect an altered metabolism of biogenic amines.

*AN, anorexia nervosa; AN-WR, previous diagnosis of anorexia nervosa, weight-restored; BP, blood pressure; BPV, blood pressure variability; BRS, baroreflex sensitivity; DBP, diastolic blood pressure; HC, healthy controls; LF, low frequency; NE, noradrenaline; NR, not reported; NS, not significant; PNS, parasympathetic nervous system; SBP, systolic blood pressure; SNS, sympathetic nervous system*.

#### (i) Current AN

Thirteen studies reported basal NE or MHPG levels in individuals with a current diagnosis of AN. Of these studies, 11 reported basal plasma NE levels; four reported decreased plasma NE (Gross et al., 1979; Luck et al., 1983; Pirke et al., 1992; D'Andrea et al., 2008), one reported increased plasma NE (Van Binsbergen et al., 1991) and six reported no difference in basal plasma NE (Lesem et al., 1989; Kaye et al., 1990; Bartak et al., 2004; Nedvidkova et al., 2004; Dostalova et al., 2007; Lechin et al., 2010), as compared to controls. Lechin et al. (2010) proposed that individuals with AN present with adrenal sympathetic overactivity, as evidenced by the low NE: adrenaline plasma ratio, yet did not directly compare NE levels to controls. Beta-adrenergic receptor activity was assessed by Kaye et al. (1990) who found an erratic response to increasing doses of isoproterenol in individuals with AN, as compared with controls, proposing that altered regulation of presynaptic adrenoreceptors may account for the discrepancy in assessments of NE levels across studies.

Two studies assessed adipose tissue levels of NE and both reported increased NE (Bartak et al., 2004; Nedvidkova et al., 2004). Two studies assessed urinary NE levels and both found decreased urinary NE, as compared to normal weight controls (De Rosa et al., 1983; Van Binsbergen et al., 1991) and lean controls (Van Binsbergen et al., 1991), despite one also reporting increased plasma NE levels (Van Binsbergen et al., 1991). Three studies assessed urinary excretion levels of MHPG; in two, MHPG levels were decreased in individuals with AN (Gross et al., 1979; Riederer et al., 1982) and in the third, there was no difference to controls (Van Binsbergen et al., 1991).

#### (ii) Weight-Restored AN

Six studies reported basal NE or MHPG levels in individuals with a previous diagnosis of AN (Gross et al., 1979; Riederer et al., 1982; Kaye et al., 1985, 1990; Lesem et al., 1989; Pirke et al., 1992). Five studies reported plasma NE levels; two of which reported decreased NE (Kaye et al., 1985; Pirke et al., 1992) and three reported no difference to controls (Gross et al., 1979; Lesem et al., 1989; Kaye et al., 1990). Two studies reported urinary MHPG levels and both found them to be comparable to control levels (Gross et al., 1979; Riederer et al., 1982) whereas one study assessed plasma MHPG, which was decreased in AN-WR participants (Kaye et al., 1985).

### Skin Conductance Level and Pupil Response

Four studies reported skin conductance level (SCL) as an outcome measure in individuals with a current diagnosis of AN (see Table 6); two reported decreased SCL (Abell et al., 1987; Palomba et al., 2017) and two reported no difference in SCL compared to controls (Calloway et al., 1983; Léonard et al., 1998).

**Table 6:** Skin conductanc and pupil response, as compared to controls.

**References**	**Variables assessed**	**Group**	**SNS**	**PNS**	**Outcome**
Abell et al. (1987)	SCL	AN	↓		Patients with AN demonstrated a decreased SNS in response to cold, compared to HCs
Calloway et al. (1983)	SCL	AN-R	NS		No difference between AN and HC in skin conductance. There was heterogeneity within the patient groups whereby patients with BN and AN-BP showed fewer spontaneous fluctuations and were faster to habituate than AN-R patients; emphasizing the similarity between BN and AN-BP
		AN-BP	NS		
Bar et al. (2006)	PLR	AN (T1)	↓	↑	Pupillary diameter, which reflects SNS was decreased, whereas the relative amplitude as a parameter resembling PNS was increased in individuals with AN in the acute stage but normalized after weight restoration
		AN-WR (T2)	NS	NS	
		AN-WR (T3)	NS	NS	
Léonard et al. (1998)	SCL	AN	NS		There was no difference between AN and HCs in baseline skin conductance but meal intake induced significantly higher increase in SCL in individuals with AN, compared to HCs. The SCL was negatively correlated with BMI and positively correlated with anxiety, depression and ED psychopathology.
Palomba et al. (2017)	SCL	AN	↓		Individuals with AN demonstrated reduced resting SCL, which was independent from BMI but negatively correlated with metacognitive scale (negative beliefs about thoughts in general). Dysfunctional metacognitions about worry might yield also a reduced sympathetic activity.

*AN, anorexia nervosa; AN-BP, anorexia nervosa, binge-purge subtype; AN-R, anorexia nervosa, restricting subtype; BMI, body mass index; SCL, skin conductance level; ED, eating disorder; HC, healthy controls; NS, not significant; PLR, pupillometry response; PNS, parasympathetic nervous system; SNS, sympathetic nervous system*.

The only study that assessed pupil response (PLR) found decreased PLR response in individuals with a current diagnosis of AN, which did not persist after weight restoration (Bar et al., 2006).

## Discussion

The current review provides the first synthesis of investigations into ANS function in individuals with AN and those who have a previous diagnosis and have achieved weight restoration. The assessment of ANS function across modalities is discussed below.

### Heartrate Variability

The majority of studies that assessed HRV in the time domain demonstrated increased beat-to-beat variability in HR in individuals with a current diagnosis of AN, consistent with a recent review (Peyser et al., 2020). Moreover, increased time domain HRV parameters were demonstrated in patients with AN when compared to lean controls (Petretta et al., 1997; Galetta et al., 2003). The studies that reported decreased time domain HRV presented some methodological limitations. One did not specify duration of AN and stated that participants had recently started various antidepressant and antipsychotic agents (Russell et al., 2008), which have been associated with decreased HRV (Licht et al., 2010), another did not report the length of HRV assessment (Lachish et al., 2009) and the third reported results from a small sample size of six patients (Melanson et al., 2004). Following weight restoration, one reported no difference to controls and the other reported decreased HRV, yet did not report the HRV assessment length (Lachish et al., 2009). Therefore, based on the current review results, beat-to-beat variability in HR is increased in the acute state of AN, which does not continue following weight restoration.

Assessment of HRV in the frequency domain, specifically in the LF and HF frequency bands, trended toward increased HF and decreased LF which was reflected in a trend toward decreased LF/HF ratios in patients with a current diagnosis of AN. Assessment of HRV in the frequency domain in WR participants primarily suggested normalization of HRV, with either no difference or levels trending toward controls. Akin to HRV assessed in the time domain, the acute state of AN is marked by increased parasympathetic activity and decreased sympathetic activity in the frequency domain, which appears to normalize following weight restoration.

Non-linear analysis of HRV was also assessed to provide a measure of complexity (α), or randomness, in heart period series that has been demonstrated to be reduced in individuals with congestive heart failure (Peng et al., 1995) and a prognostic indicator of cardiac mortality (Huikuri et al., 2000). Decreased α values were demonstrated in individuals with a current diagnosis of AN (Ishizawa et al., 2008; Vigo et al., 2008) and in those with a shorter duration of AN (termed “acute”) (Platisa et al., 2006), reflective of HRV patterns seen in patients with heart failure, which was postulated to be a mechanism of cardiac autonomic dysfunction and sudden death in AN (Vigo et al., 2008).

While the majority of studies indicated concordant results in HRV assessment, discrepancies are likely to be due in part to the duration of AN, the potential for comorbid conditions to impact HRV and the assessment methodology. The impact of chronicity (or duration of AN) was repeatedly highlighted as a distinguishing feature of HRV profile HRV (Platisa et al., 2006; Nakai et al., 2015). It was suggested that the HRV profile was so distinct between initial and chronic stages of illness that it could be used to distinguish between phases of illness, whereby initial starvation is typified by increased parasympathetic activity (increased HF) and an extended duration of illness was characterized by increased sympathetic activity (LF) (Petretta et al., 1997; Melanson et al., 2004; Roche et al., 2004; Platisa et al., 2006; Nakai et al., 2015). A single study found contrasting results (a positive correlation between increased illness duration and HF but a negative correlation between duration and LF), yet did not specify illness duration, therefore potential extrapolation is uncertain (Wu et al., 2004). A tentative conclusion may be that the relative increase or decrease in HF and LF is dependent on duration of AN. However, further investigation is required to confirm this hypothesis.

In addition to duration of illness, another potential influence on HRV that must be taken into account is the potential impact of comorbid psychiatric conditions on HRV parameters (Shinba et al., 2008). Anxiety and stress have been demonstrated to increase sympathetic activity (Lucini et al., 2002) and evoke cardiac vagal withdrawal, a physiological response thought to be related to the hypersensitivity engendered in anxiety disorders (for a review on the topic, see Friedman, 2007). Similarly, decreased HRV has frequently been associated with depression (independent from cardiovascular disease) (Musselman et al., 1998; Kemp et al., 2010) and antidepressant use (Licht et al., 2010; Michael and Kaur, 2021). Given that the majority of studies did not specify comorbid psychiatric conditions or psychoactive medication use, the impact of these in the current review cannot be ascertained. There is a wide literature on the influence of psychological state on HRV (Thayer et al., 2012), with common reference to Porges' polyvagal theory which stipulates that HRV is associated with experience and expression of social and emotional behavior (Porges, 2007). Given the high rate of comorbid psychiatric disorders in individuals with AN (O'Brien and Vincent, 2003), it may be difficult to extrapolate reliably, the influence of AN alone on HRV.

Further consideration must be applied when considering the HRV assessment methodology. Assessments of HRV in the current review were derived from both ambulatory recordings and short-term recordings of varying length. While HRV analyses of different lengths of time are generally closely correlated (Costa et al., 1994), results between short-term and ambulatory recordings can differ (Li et al., 2019) and should not be compared (Task Force of The European Society of Cardiology The North American Society of Pacing Electrophysiology, 1996). Indeed, the only study that assessed both short-term and ambulatory HRV in the current review reported no difference in short-term HRV but decreased HRV over long-term recordings (Melanson et al., 2004).

A separate consideration is concern over whether HRV is a reflection of the autonomic state of the entire body or the regulation of the sinoatrial node alone (Hayano and Yuda, 2019). The use of HRV as a sole index of ANS activity is potentially problematic given that frequency domain analysis of HRV reportedly over-simplifies the non-linear interactions between the SNS and PNS (Billman, 2013). While HRV provides some insight into vagal activity, it has the disadvantage of giving a poor indication of sympathetic activity (Esler and Lambert, 2003; Billman, 2013). Indeed, LF heart rate spectral power (often interpreted as sympathetic activity) has been demonstrated as unrelated to direct assessments of sympathetic activity, such as NE spillover, MSNA (Kingwell et al., 1994), and cardiac sympathetic innervation quantified by positron emission tomographic neuroimaging (Rahman et al., 2011). Moreover, in the current review, 17 out of the 25 studies that assessed HRV did not use any other method to assess autonomic function in individuals with AN, a limitation underscored by Ishizawa et al. (2008) and Takimoto et al. (2014).

Overall, the assessments of HRV indicated alterations in autonomic regulation of heart rate in AN characterized by increased heart rate variance and increased vagal activity. While persistent sympathetic excitation and depressed vagal activity are associated with ventricular arrhythmias and sudden cardiac death (Task Force of The European Society of Cardiology The North American Society of Pacing Electrophysiology, 1996), the implications of persistent vagal activation and autonomic dysregulation remain unclear. However, there have been indications of increased parasympathetic activity and autonomic dysregulation at the onset of acute myocardial infarction (Webb et al., 1972), with the suggestion that autonomic dysregulation is a risk factor for sudden cardiac death in individuals with amyotrophic lateral sclerosis (Asai et al., 2007). Therefore, it remains to be determined whether consistent elevation of HRV and increased vagal modulation of cardiac control represent cardiovascular risk for individuals with AN.

### Orthostatic Response, Blood Pressure Variability, and Baroreflex Sensitivity

Assessment of the physiological response to an orthostatic challenge can provide powerful insight into cardiac autonomic regulation. During a head-up tilt, the resultant peripheral venous pooling and decreased cardiac output triggers stimulation of aortic, carotid and cardiopulmonary baroreceptors, resulting in increased sympathetic outflow and inhibition of parasympathetic activity in healthy individuals (Ramírez-Marrero et al., 2007).

Observations that assessed the change in BP from a supine to upright position were limited; while BP response to orthostasis was blunted in individuals with AN in one study (Casu et al., 2002), it did not differ from controls in others (Lechin et al., 2010; Takimoto et al., 2014). Multiple studies compared absolute BP levels between AN and HC groups during an orthostatic challenge; a methodology which is limited in providing an indication of autonomic regulation given that BP is principally decreased in individuals with AN. However, assessments of HRV, BPV and adrenergic response to orthostasis revealed that individuals with AN failed to exhibit an increased sympathetic response to a head-up tilt. While a normal response is demonstrated by a decrease in the HF and increase in LF components of HRV and BPV, these reflex mechanisms were not seen in individuals with AN (Casu et al., 2002; Murialdo et al., 2007; Takimoto et al., 2014). Furthermore, individuals with AN did not demonstrate increased adrenergic outflow during a change in position (Gross et al., 1979; Lechin et al., 2010), yet were comparable to controls after weight restoration (Gross et al., 1979; Lesem et al., 1989).

While at rest, individuals with AN demonstrated decreased variability in BP and increased baroreflex sensitivity, further suggesting increased parasympathetic control over the heart. Together, these assessments of orthostatic response, BPV and BRS in individuals with AN demonstrate an abnormal regulation of the cardiovascular system through a failure to activate a sympathetic response and inhibit parasympathetic activity. Altered orthostatic regulation suggests that individuals with AN are at risk of a range of conditions associated with altered orthostatic regulation, such as syncope, orthostatic hypertension, and POTS (Grubb, 2005), many of which have indeed been reported in AN. Following weight-restoration, responses trended toward those of controls, reflective of the suggestion that resolution of a normal orthostatic response can determine medical stability and readiness for discharge following treatment (Shamim et al., 2003).

### Adrenergic Assessment

While many of the studies that assessed static adrenergic activity in the current review found no difference in plasma NE levels between individuals with AN and controls, there was a trend toward decreased plasma NE or MHPG levels. Decreased NE was interpreted as a chronic adaptation to malnutrition by some authors (Riederer et al., 1982; Dostalova et al., 2007), which contributed to hypothalamic dysfunction during the acute state of AN (Gross et al., 1979; De Rosa et al., 1983). Another interpretation suggested that NE levels varied over the course of treatment according to stress levels and psychological (as opposed to physical) stabilization (Lesem et al., 1989). Moreover, altered regulation of presynaptic beta-adrenoreceptors was reported, suggesting that altered noradrenergic receptor function may also be present in individuals with AN (Kaye et al., 1990).

Similarly, urinary excretion of NE and MHPG was decreased in individuals with AN compared to both normal weight (Gross et al., 1979; De Rosa et al., 1983) and lean controls (Van Binsbergen et al., 1991), which increased following treatment (Gross et al., 1979; Riederer et al., 1982). While MHPG is the major metabolite of NE in the brain, urinary MHPG is predominantly the product of peripheral SNS, rather than central nervous system NE metabolism. Given that urinary catecholamine excretion is dependent on renal function (Esler et al., 1988), which has previously been shown to be impaired in individuals with AN (Stheneur et al., 2014), interpretation of decreased urinary excretion of NE and MHPG in AN is constrained.

In contrast, assessment of NE levels in adipose tissue revealed localized elevated levels of sympathetic activity in individuals with AN, compared to controls (Bartak et al., 2004; Nedvidkova et al., 2004), despite no difference in overall plasma NE (Bartak et al., 2004). Given that local adipose tissue sympathetic activity is not a reflection of overall whole body sympathetic activity (Patel et al., 2002), an increase in localized sympathetic activity within adipose tissue was suggested to be a protective mechanism to protect fat stores from further depletion through downregulation of lipolysis (Bartak et al., 2004), a process supported by prolonged fasting models (Migliorini et al., 1997).

Each assessment of adrenergic activity in individuals with a current diagnosis of AN, and after weight restoration, provided an alternate assessment of NE presence and metabolism. Given that circulating NE levels represent a small proportion of NE secreted from nerve terminals (Grassi and Esler, 1999), it is difficult to surmise a conclusive interpretation of sympathetic activity from these results. However, there was a trend toward decreased NE levels in individuals with a current diagnosis of AN, which normalized after weight restoration.

### Skin Conductance Level and Pupillary Response

In comparison to alternate measurements of autonomic function, SCL and PLR were less commonly assessed. Notwithstanding this, reduced sympathetic activation in SCL (Abell et al., 1987; Palomba et al., 2017) and altered SCL responses between AN subtype (Calloway et al., 1983) were reported. All assessments of SCL were conducted on the palms, of which are prone to emotional sweating (Vetrugno et al., 2003). Indeed, alterations to SCL in AN were observed to be correlated with psychological factors (including anxiety and metacognitive dysfunction) (Léonard et al., 1998; Palomba et al., 2017). Given that sympathetic skin response has been demonstrated to be emotionally activated (Cheshire et al., 2020), the use of SCL to provide insight into thermoregulatory autonomic function is therefore limited.

The only study that investigated PLR found decreased sympathetic and increased parasympathetic pupil response in individuals with AN, yet only in the acute state, which normalized following weight restoration (Bar et al., 2006). Given that only a single investigation has been conducted into PLR, which identified changes in autonomic nervous system activity in individuals with AN, further investigations of this non-invasive parameter should be undertaken in future studies.

### Limitations

The purpose of the current review was to synthesize the evidence of ANS function associated with AN. Several methodological factors must be taken into account when comparing the assessments of ANS function in the current review. Given the serious nature and medical instability associated with AN, many studies utilized small sample sizes, which no doubt contributed to the lack of consistency among results in individual studies. Moreover, the studies investigating individuals with a previous diagnosis of AN included varied durations of weight restoration, precluding the ability to draw a succinct conclusion. Many studies did not detail or compare differences between restrictive and binge eating-purging subtypes of AN, therefore any differences related to specific AN behaviors cannot be determined by the current review. Future investigations into ANS function after a prolonged period of weight restoration would allow a better understanding of the impact of AN in any long-term alterations to ANS function. Similarly, delineation of AN subtype and assessment of comorbid psychiatric diagnoses in future assessments could reveal any differences in autonomic function according to subtype and comorbidities.

### Implications and Conclusion

The current review provides a synthesis of the evidence to date assessing resting autonomic function in individuals with AN, and after weight restoration. It is indicated that individuals with AN demonstrate autonomic dysregulation characterized by decreased sympathetic activity and increased parasympathetic activity as well as increased complexity of the ANS through a variety of assessment methodologies. Given the ease and convenience of HRV assessment, it is tempting to use the measure as a sole assessment of autonomic function. However, the demonstrated impact that both illness duration and psychiatric comorbidities can have on HRV infer that assessment of autonomic activity should be established via additional accompanying measures. While the duration of weight restoration in the current review was widely varied, the majority of studies to date indicated that autonomic regulation tended to normalize after weight restoration. Moreover, there has been no assessment of SNS activity in individuals with AN to date using either microneurographic measurement of muscle sympathetic nerve activity or assessment of organ-specific NE spillover; the two “preferred” assessments of human adrenergic function (Grassi and Esler, 1999).

The underlying mechanisms that contribute to the abnormalities in ANS function in acute AN remain speculative. It has been proposed that the parasympathetic dominance seen in AN is an adaptive physiological response to conserve energy in response to malnutrition (Buchhorn et al., 2016; Sachs et al., 2016; Kalla et al., 2017). However, it remains unclear whether energy preservation alone is underlying the changes in ANS function, given that the three studies that included lean control groups did not find a linear relationship between BMI and ANS function. Specifically, HRV and NE excretion in patients with AN were significantly different than both normal-weight and lean controls, who satisfied the weight, but not psychological, criterion for AN (Van Binsbergen et al., 1991; Petretta et al., 1997; Galetta et al., 2003). There is growing evidence of an intrinsic connection between the brain and the heart, including interplay between frontal-vagal (brain-heart) and depression networks (Iseger et al., 2020), that purportedly contributes to cardiovascular disease (Makovac et al., 2017). Given the demonstrated dysregulation of other neural regulatory systems in AN [including dopaminergic and serotonergic systems, which are thought to contribute to both physiological and psychological traits seen in AN (Kaye et al., 2005; Fladung et al., 2010)], there may be central dysregulation of ANS networks in AN, yet this remains putative.

The implications of the current review are that increased vagal activity is likely to underlie the widespread bradycardia in individuals with AN. Moreover, inhibited SNS activation during orthostasis would result in insufficient blood flow to organs and contribute to episodes of syncope. Less clear are the implications of the increased autonomic complexity demonstrated by HRV and BRS parameters. While cardiovascular disease is commonly associated with *sympathetic* overactivity (Malpas, 2010), the consequences of sustained *parasympathetic* overactivity and autonomic dysregulation are yet to be determined. It remains to be ascertained whether the autonomic dysregulation indicated in individuals with AN contributes to the widespread cardiovascular complications.

This review has demonstrated that autonomic dysregulation is indicated in individuals with AN, yet there have been no thorough assessments of autonomic function utilizing multiple methodologies. Due to the variability in both methodology and quality of assessments to date, conclusions drawn from these data should be interpreted with caution. Furthermore, in order to determine the association between autonomic dysregulation and widespread cardiovascular complications in AN conclusively, future investigations should employ a variety of assessments of autonomic function in conjunction with markers of cardiovascular risk. It will also be important to assess the impact of comorbid psychiatric conditions and duration of illness in order to conclusively establish the nature of autonomic (dys)function in AN. Similarly, future investigations in individuals with an extended duration of weight restoration are still required. Determination of autonomic function through a variety of assessment methodologies in individuals with a current, and previous, diagnosis of AN alongside assessments of cardiovascular risk will aid in determining the contributing factors to cardiovascular complications. This will allow clinicians to identify individuals at risk and aid in the prevention, treatment and development of interventions to reduce the inadvertent mortality rate of AN.

## Data Availability Statement

The original contributions presented in the study are included in the article/Supplementary Material, further inquiries can be directed to the corresponding author/s.

## Author Contributions

ZJ conceived the project and drafted the manuscript. HW conducted the search. ZJ, EL, and NE conducted the study selection. ZJ, NE, AP, DC, and EL conducted the data extraction and risk of bias. All authors designed and approved the protocol, reviewed, and approved.

## Conflict of Interest

The authors declare that the research was conducted in the absence of any commercial or financial relationships that could be construed as a potential conflict of interest.
