# Supplementary material for: Autonomic Nervous System Function in Anorexia Nervosa: A Systematic Review
Source: Front Neurosci. 2021 Jun 28;15:682208. doi: 10.3389/fnins.2021.682208 (PMC8273292; doi:10.3389/fnins.2021.682208)
Supplement: Supplementary file 1 [file Data_Sheet_1.docx]

**Appendix 1. Search strategies**

**Ovid MEDLINE(R) ALL 1946 to November 03, 2020**

1. Anorexia Nervosa/ or Anorexia/ or "Feeding and Eating Disorders"/
2. (anorex* or eating disorder*).ti,ab,kw.
3. 1 or 2
4. autonomic nervous system/ or autonomic pathways/ or autonomic fibers, postganglionic/ or parasympathetic fibers, postganglionic/ or sympathetic fibers, postganglionic/ or autonomic fibers, preganglionic/ or celiac plexus/ or submucous plexus/ or vagus nerve/ or ganglia, autonomic/ or ganglia, sympathetic/ or stellate ganglion/ or superior cervical ganglion/ or parasympathetic nervous system/ or ganglia, parasympathetic/ or glossopharyngeal nerve/ or sympathetic nervous system/ or splanchnic nerves/ or vasomotor system/ or pressoreceptors/
5. autonomic nervous system diseases/ or autonomic dysreflexia/ or reflex sympathetic dystrophy/ or dysautonomia, familial/ or exp multiple system atrophy/ or exp orthostatic intolerance/ or pure autonomic failure/ or sweating, gustatory/
6. (autonomic or celiac plexus or enteric nervous system* or parasympathetic gangli*).ti,ab,kw.
7. (parasympathetic nervous system* or postganglionic parasympathetic fib* or postganglionic sympathetic fib* or pressoreceptor*).ti,ab,kw.
8. (splanchnic nerve* or stellate gangli* or superior cervical gangli* or submucous plexus or sympathetic gangli* or sympathetic nervous system*).ti,ab,kw.
9. (vagus nerve* or vasomotor system* or vegetative nervous system* or visceral nervous system*).ti,ab,kw.
10. (reflex sympathetic dystrophy or familial dysautonomia or multiple system atrophy or orthostatic intolerance* or gustatory sweating).ti,ab,kw.
11. catecholamines/ or norepinephrine/ or droxidopa/ or nordefrin/ or normetanephrine/
12. (catecholamine* or droxidopa or noradrenaline* or nordefrin or norepinephrine* or normetanephrine*).ti,ab,kw.
13. (dysautonomi* or orthostatic intolerance or adrenergic transmission or parasympathetic function or parasympathetic tone or sympathetic function or sympathetic reflex or sympathetic tone).ti,ab,kw.
14. ((heart rate adj1 varia*) or (blood pressure adj1 varia*) or baroreflex sensitivit* or microneurography or sudomotor or pupillometry).ti,ab,kw.
15. *blood pressure/ or *heart rate/
16. 4 or 5 or 6 or 7 or 8 or 9 or 10 or 11 or 12 or 13 or 14 or 15
17. 3 and 16
18. exp animals/ not humans/
19. (mice or mouse or rat or rats or animal* or horse* or rodent*).ti.
20. 18 or 19
21. 17 not 20
22. limit 21 to english language

**Embase 1974 to 2020 November 03 (Ovid)**

1. anorexia nervosa/ or anorexia/ or eating disorder/
2. (anorex* or eating disorder*).ti,ab.
3. 1 or 2
4. autonomic nervous system/ or autonomic ganglion/ or celiac plexus/ or parasympathetic ganglion/ or parasympathetic nerve/ or parasympathetic nerve cell/ or vagus nerve/
5. catecholaminergic system/ or noradrenergic system/ or noradrenergic nerve/ or noradrenergic nerve cell/ or stellate ganglion/ or sympathetic ganglion/ or sympathetic innervation/ or sympathetic nerve/ or sympathetic nerve cell/ or catecholamine nerve cell/
6. catecholamine/ or noradrenalin/ or catecholamine blood level/ or noradrenalin blood level/ or catecholamine brain level/ or catecholamine depletion/ or catecholamine excretion/ or catecholamine metabolism/ or catecholamine synthesis/ or catecholamine release/ or noradrenalin release/ or catecholamine transporter/ or catecholamine uptake/ or catecholamine urine level/ or noradrenalin urine level/
7. (autonomic or celiac plexus or enteric nervous system* or parasympathetic gangli*).ti,ab.
8. (parasympathetic nervous system* or postganglionic parasympathetic fib* or postganglionic sympathetic fib* or pressoreceptor*).ti,ab.
9. (splanchnic nerve* or stellate gangli* or superior cervical gangli* or submucous plexus or sympathetic gangli* or sympathetic nervous system*).ti,ab.
10. (vagus nerve* or vasomotor system* or vegetative nervous system* or visceral nervous system*).ti,ab.
11. (catecholamine* or droxidopa or noradrenaline* or nordefrin or norepinephrine* or normetanephrine*).ti,ab.
12. autonomic dysfunction/ or *autonomic neuropathy/ or *cardiac autonomic neuropathy/ or *dysautonomia/ or exp *orthostatic intolerance/ or *pure autonomic failure/
13. autonomic nervous system function/ or exp *adrenergic transmission/ or exp *autonomic innervation/ or *parasympathetic function/ or *parasympathetic tone/ or *sympathetic function/ or *sympathetic reflex/ or *sympathetic tone/
14. (dysautonomi* or orthostatic intolerance or adrenergic transmission or parasympathetic function or parasympathetic tone or sympathetic function or sympathetic reflex or sympathetic tone).ti,ab.
15. ((heart rate adj1 varia*) or (blood pressure adj1 varia*) or baroreflex sensitivit* or microneurography or sudomotor or pupillometry).ti,ab.
16. heart rate variability/ or blood pressure variability/
17. 4 or 5 or 6 or 7 or 8 or 9 or 10 or 11 or 12 or 13 or 14 or 15 or 16
18. 3 and 17
19. exp animal/ not human/
20. (mice or mouse or rat or rats or animal* or horse* or rodent*).ti.
21. 19 or 20
22. 18 not 21
23. limit 22 to english language

**Ovid Emcare 1995 to 2020 Week 44**

1. anorexia nervosa/ or anorexia/ or eating disorder/
2. (anorex* or eating disorder*).ti,ab.
3. 1 or 2
4. autonomic nervous system/ or autonomic ganglion/ or celiac plexus/ or parasympathetic ganglion/ or parasympathetic nerve/ or parasympathetic nerve cell/ or vagus nerve/
5. catecholaminergic system/ or noradrenergic system/ or noradrenergic nerve/ or noradrenergic nerve cell/ or stellate ganglion/ or sympathetic ganglion/ or sympathetic innervation/ or sympathetic nerve/ or sympathetic nerve cell/ or catecholamine nerve cell/
6. catecholamine/ or noradrenalin/ or catecholamine blood level/ or noradrenalin blood level/ or catecholamine brain level/ or catecholamine depletion/ or catecholamine excretion/ or catecholamine metabolism/ or catecholamine synthesis/ or catecholamine release/ or noradrenalin release/ or catecholamine transporter/ or catecholamine uptake/ or catecholamine urine level/ or noradrenalin urine level/
7. (autonomic or celiac plexus or enteric nervous system* or parasympathetic gangli*).ti,ab.
8. (parasympathetic nervous system* or postganglionic parasympathetic fib* or postganglionic sympathetic fib* or pressoreceptor*).ti,ab.
9. (splanchnic nerve* or stellate gangli* or superior cervical gangli* or submucous plexus or sympathetic gangli* or sympathetic nervous system*).ti,ab.
10. (vagus nerve* or vasomotor system* or vegetative nervous system* or visceral nervous system*).ti,ab.
11. (catecholamine* or droxidopa or noradrenaline* or nordefrin or norepinephrine* or normetanephrine*).ti,ab.
12. autonomic dysfunction/ or *autonomic neuropathy/ or *cardiac autonomic neuropathy/ or *dysautonomia/ or exp *orthostatic intolerance/ or *pure autonomic failure/
13. autonomic nervous system function/ or exp *adrenergic transmission/ or exp *autonomic innervation/ or *parasympathetic function/ or *parasympathetic tone/ or *sympathetic function/ or *sympathetic reflex/ or *sympathetic tone/
14. (dysautonomi* or orthostatic intolerance or adrenergic transmission or parasympathetic function or parasympathetic tone or sympathetic function or sympathetic reflex or sympathetic tone).ti,ab.
15. ((heart rate adj1 varia*) or (blood pressure adj1 varia*) or baroreflex sensitivit* or microneurography or sudomotor or pupillometry).ti,ab.
16. heart rate variability/ or blood pressure variability/
17. 4 or 5 or 6 or 7 or 8 or 9 or 10 or 11 or 12 or 13 or 14 or 15 or 16
18. 3 and 17
19. exp animal/ not human/
20. (mice or mouse or rat or rats or animal* or horse* or rodent*).ti.
21. 19 or 20
22. 18 not 21
23. limit 22 to english language

**APA PsycInfo 1806 to October Week 4 2020 (Ovid)**

1. anorexia nervosa/ or eating disorders/
2. (anorex* or eating disorder*).ti,ab.
3. 1 or 2
4. autonomic nervous system/ or autonomic ganglia/ or parasympathetic nervous system/ or vagus nerve/ or sympathetic nervous system/ or autonomic nervous system disorders/
5. catecholamines/ or norepinephrine/
6. (autonomic or celiac plexus or enteric nervous system* or parasympathetic gangli*).ti,ab.
7. (parasympathetic nervous system* or postganglionic parasympathetic fib* or postganglionic sympathetic fib* or pressoreceptor*).ti,ab.
8. (splanchnic nerve* or stellate gangli* or superior cervical gangli* or submucous plexus or sympathetic gangli* or sympathetic nervous system*).ti,ab.
9. (vagus nerve* or vasomotor system* or vegetative nervous system* or visceral nervous system*).ti,ab.
10. (catecholamine* or droxidopa or noradrenaline* or nordefrin or norepinephrine* or normetanephrine*).ti,ab.
11. (dysautonomi* or orthostatic intolerance or adrenergic transmission or parasympathetic function or parasympathetic tone or sympathetic function or sympathetic reflex or sympathetic tone).ti,ab.
12. ((heart rate adj1 varia*) or (blood pressure adj1 varia*) or baroreflex sensitivit* or microneurography or sudomotor or pupillometry).ti,ab.
13. heart rate variability/
14. 4 or 5 or 6 or 7 or 8 or 9 or 10 or 11 or 12 or 13
15. 3 and 14
16. animals/ not humans/
17. (mice or mouse or rat or rats or animal* or horse* or rodent*).ti.
18. 16 or 17
19. 15 not 18
20. limit 19 to english language

**Ovid Nursing Database 1946 to October Week 4 2020**

1. anorexia nervosa/ or eating disorders/ or anorexia/
2. (anorex* or eating disorder*).ti,ab.
3. 1 or 2
4. exp autonomic nervous system/ or autonomic nervous system diseases/ or autonomic dysreflexia/
5. catecholamines/ or norepinephrine/
6. (autonomic or celiac plexus or enteric nervous system* or parasympathetic gangli*).ti,ab.
7. (parasympathetic nervous system* or postganglionic parasympathetic fib* or postganglionic sympathetic fib* or pressoreceptor*).ti,ab.
8. (splanchnic nerve* or stellate gangli* or superior cervical gangli* or submucous plexus or sympathetic gangli* or sympathetic nervous system*).ti,ab.
9. (vagus nerve* or vasomotor system* or vegetative nervous system* or visceral nervous system*).ti,ab.
10. (catecholamine* or droxidopa or noradrenaline* or nordefrin or norepinephrine* or normetanephrine*).ti,ab.
11. (dysautonomi* or orthostatic intolerance or adrenergic transmission or parasympathetic function or parasympathetic tone or sympathetic function or sympathetic reflex or sympathetic tone).ti,ab.
12. ((heart rate adj1 varia*) or (blood pressure adj1 varia*) or baroreflex sensitivit* or microneurography or sudomotor or pupillometry).ti,ab.
13. heart rate variability/
14. 4 or 5 or 6 or 7 or 8 or 9 or 10 or 11 or 12 or 13
15. 3 and 14
16. exp animals/
17. (mice or mouse or rat or rats or animal* or horse* or rodent*).ti.
18. 16 or 17
19. 15 not 18
20. limit 19 to english language

**CINAHL (EBSCOhost)**

1. (MH "Anorexia Nervosa") OR (MH "Anorexia") OR (MH "Eating Disorders")
2. anorex* OR "eating disorder*"
3. S1 OR S2
4. (MH "Autonomic Nervous System+") OR (MH "Autonomic Nervous System Diseases")
5. (MH "Catecholamines") OR (MH "Norepinephrine")
6. "autonomic" OR "celiac plexus" OR "enteric nervous system*" OR "parasympathetic gangli*"
7. "parasympathetic nervous system*" OR "postganglionic parasympathetic fib*" OR "postganglionic sympathetic fib*" OR pressoreceptor*
8. "splanchnic nerve*" OR "stellate gangli*" OR "superior cervical gangli*" OR "submucous plexus" OR "sympathetic gangli*" OR "sympathetic nervous system*"
9. "vagus nerve*" OR "vasomotor system*" OR "vegetative nervous system*" OR "visceral nervous system*"
10. catecholamine* OR droxidopa OR noradrenaline* OR nordefrin OR norepinephrine* OR normetanephrine*
11. dysautonomi* OR "orthostatic intolerance" OR "adrenergic transmission" OR "parasympathetic function" OR "parasympathetic tone" OR "sympathetic function" OR "sympathetic reflex" OR "sympathetic tone"
12. (MH "Heart Rate Variability")
13. ("heart rate" N1 varia*) OR ("blood pressure" N1 varia*) OR "baroreflex sensitivit*" OR microneurography OR sudomotor OR pupillometry
14. S4 OR S5 OR S6 OR S7 OR S8 OR S9 OR S10 OR S11 OR S12 OR S13
15. S3 AND S14 [limit to English language]

**Cochrane Library**

1. [mh ^"Anorexia Nervosa"] OR [mh ^Anorexia] OR [mh ^"Feeding and Eating Disorders"]
2. (anorex* OR "eating disorder*"):ti,ab
3. #1 OR #2
4. [mh ^"autonomic nervous system"] OR [mh ^"autonomic pathways"] OR [mh ^"autonomic fibers, postganglionic"] OR [mh ^"parasympathetic fibers, postganglionic"] OR [mh ^"sympathetic fibers, postganglionic"] OR [mh ^"autonomic fibers, preganglionic"] OR [mh ^"celiac plexus"] OR [mh ^"submucous plexus"] OR [mh ^"vagus nerve"] OR [mh ^"ganglia, autonomic"] OR [mh ^"ganglia, sympathetic"] OR [mh ^"stellate ganglion"] OR [mh ^"superior cervical ganglion"] OR [mh ^"parasympathetic nervous system"] OR [mh ^"ganglia, parasympathetic"] OR [mh ^"glossopharyngeal nerve"] OR [mh ^"sympathetic nervous system"] OR [mh ^"splanchnic nerves"] OR [mh ^"vasomotor system"] OR [mh ^pressoreceptors]
5. (autonomic OR "celiac plexus" OR "enteric nervous system*" OR "parasympathetic gangli*"):ti,ab
6. ("parasympathetic nervous system*" OR "postganglionic parasympathetic fib*" OR "postganglionic sympathetic fib*" OR pressoreceptor*):ti,ab
7. ("splanchnic nerve*" OR "stellate gangli*" OR "superior cervical gangli*" OR "submucous plexus" OR "sympathetic gangli*" OR "sympathetic nervous system*"):ti,ab
8. ("vagus nerve*" OR "vasomotor system*" OR "vegetative nervous system*" OR "visceral nervous system*"):ti,ab
9. [mh ^catecholamines] OR [mh ^norepinephrine] OR [mh ^droxidopa] OR [mh ^nordefrin] OR [mh ^normetanephrine]
10. (catecholamine* OR droxidopa OR noradrenaline* OR nordefrin OR norepinephrine* OR normetanephrine*):ti,ab
11. (dysautonomi* OR "orthostatic intolerance" OR "adrenergic transmission" OR "parasympathetic function" OR "parasympathetic tone" OR "sympathetic function" OR "sympathetic reflex" OR "sympathetic tone"):ti,ab
12. (("heart rate" NEAR/1 varia*) OR ("blood pressure" NEAR/1 varia*) OR "baroreflex sensitivit*" OR microneurography OR sudomotor OR pupillometry):ti,ab
13. [mh ^"blood pressure"] OR [mh ^"heart rate"]
14. #4 OR #5 OR #6 OR #7 OR #8 OR #9 OR #10 OR #11 OR #12 OR #13
15. #3 AND #14

**Health Collection, Humanities & Social Sciences Collection (Informit)**

( autonomic OR parasympathetic OR sympathetic or catecholamine* or norepinephrine*) AND ( anorexi* OR (eating disorder*))

**Clinicaltrials.gov**

autonomic OR parasympathetic OR sympathetic OR catecholamine OR norepinephrine | Anorexia Nervosa
