# Supplementary material for: Autonomic Nervous System Function in Anorexia Nervosa: A Systematic Review
Source: Front Neurosci. 2021 Jun 28;15:682208. doi: 10.3389/fnins.2021.682208 (PMC8273292; doi:10.3389/fnins.2021.682208)
Supplement: Supplementary file 2 [file Data_Sheet_2.docx]

**Appendix 2. Modified Newcastle-Ottawa Quality Assessment Scale for Cohort/Case control studies**

Note: A study can be awarded a maximum of one star for each numbered item within the Selection and Outcome categories. A maximum of two stars can be given for Comparability

**SELECTION**

1. AN group was adequately defined
   1. diagnostic classification reference with inclusion/exclusion criteria clearly defined *
   2. diagnostic classification reference only
2. Representative control selection
   1. Community controls *
   2. Hospital controls/selected group of controls eg nurses/family members
   3. No description of the derivation of controls
3. Definition of controls
   1. No history of disease & inclusion/exclusion criteria adequately defined *
   2. No description

**COMPARABILITY**

1. Comparability of groups on the basis of the design or analysis
   1. Study controlled for BMI *
   2. Study controlled for additional factors *

**OUTCOME**

1. Assessment of outcome was adequately conducted
   1. Appropriate guidelines and methodology specified *
   2. Inadequate/no description
2. Were the methods (including statistical methods) sufficiently described to be able them to be repeated?
   1. Yes *
   2. No
3. Were the basic data adequately presented
   1. Data were clearly and adequately presented including confidence intervals where appropriate *
   2. Data were not adequately presented
4. Were the authors’ discussions and conclusions justified by the results?
   1. Yes *
   2. No
5. Were the limitations of the study discussed?
   1. Yes *
   2. No

Risk of bias in studies was assessed by using a modified version of the Newcastle-Ottawa Quality Assessment Scale. The studies were scored out of 10; 6 or more points was considered to be at low risk of bias, studies that scored 4-5 points to be at moderate risk, and those with less than 4 points to be at high risk of bias.
