# Supplementary material for: Autonomic Nervous System Function in Anorexia Nervosa: A Systematic Review
Source: Front Neurosci. 2021 Jun 28;15:682208. doi: 10.3389/fnins.2021.682208 (PMC8273292; doi:10.3389/fnins.2021.682208)
Supplement: Supplementary file 3 [file Data_Sheet_3.docx]

**Appendix 3. HRV risk of bias/quality assessment**

**APPROPRIATE SELECTION OF PARTICIPANTS**

(1) Has the diagnosis of the cases been reliably assessed and validated?

According to international criteria by a physician (2)

According to international criteria, assessor not clearly established (1)

Self-report or not clearly stated (0)

(2) Were recruitment details of control group and method used to rule out psychiatric illness specified?

Recruitment details AND methods used to rule out psychiatric illness specified (2)

Only recruitment details OR methods used to rule out psychiatric illness specified (1)

Not clearly stated (0)

(3) Is the population deﬁned with in- and exclusion-criteria?

Medication use, somatic morbidity, psychiatric morbidity, 3 stated (2)

Medication use, somatic morbidity, psychiatric morbidity 1-2 stated (1)

None stated or not clearly stated (0)

(4) Are disorder characteristics presented (length and severity (BMI) of disorder)?

Duration of disease and severity of disorder (BMI) is stated (2)

Only duration or only severity (BMI) is stated (1)

None stated (0)

**APPROPRIATE QUANTIFICATION OF HEART RATE VARIABILITY (HRV)**

(5) Are hardware/software details of collection stated?

Brand and electrode configuration stated (2)

Brand or electrode configuration stated (1)

Not clearly stated (0)

(6) Are HRV collection details provided?

Raw sampling rate, length of data collection, time of day, filtering, participant posture and instructions stated, 5-6 stated (2)

Raw sampling rate, length of data collection, time of day, filtering, participant posture and instructions stated, 3-4 stated (1)

Raw sampling rate, length of data collection, time of day, filtering, participant posture and instructions stated, <3 stated (0)

(7) Are methods for data analysis and cleaning of HRV clearly stated?

IBI calculation, resampling method, artefact identification, artefact cleaning methods, reasons for loss, 4-5 stated (2)

IBI calculation, resampling method, artefact identification, artefact cleaning methods, reasons for loss, 2-3 stated (1)

IBI calculation, resampling method, artefact identification, artefact cleaning methods, reasons for loss, 1 or none stated (0)

(8) Are methods for HRV calculation clearly stated?

Metrics used, software/script used, log transformation (if applicable), 1-3 stated (2)

No HRV calculation methods stated (0)

(9) Is outcome HRV clearly described and presented?

Appropriate units (absolute units or normalized units) and measures of dispersion stated (2)

Only appropriate units but no measures of dispersion stated (1)

Outcome not clearly stated (0)

**APPROPRIATE CONTROL FOR CONFOUNDING**

(10) Are potential confounders assessed^a^?

Age, gender, body mass index, smoking, depression, anxiety, medication use, 5–7 stated (2)

Age, gender, body mass index, smoking, depression, anxiety, medication use, 3–4 stated (1)

Age, gender, body mass index, smoking, depression, anxiety, medication use, 1–2 or none stated (0)

(11) Are the analyses adjusted for potential confounders^b^?

Age, gender, body mass index, smoking, depression, anxiety, medication use, 5–7 stated (2)

Age, gender, body mass index, smoking, depression, anxiety, medication use, 3–4 stated (1)

Age, gender, body mass index, smoking, depression, anxiety, medication use, 1–2 or none stated (0)

^a^ In case of exclusion at item 3, consider confounder as assessed. ^b^ In case of exclusion at item 3 or no signiﬁcant difference between cases and controls at item 7 consider confounder as adjusted for.
