# Supplementary material for: Autonomic Nervous System Function in Anorexia Nervosa: A Systematic Review
Source: Front Neurosci. 2021 Jun 28;15:682208. doi: 10.3389/fnins.2021.682208 (PMC8273292; doi:10.3389/fnins.2021.682208)
Supplement: Supplementary file 4 [file Data_Sheet_4.docx]

**Appendix 4. Study quality assessment using the Modified Newcastle-Ottawa Quality Assessment Scale**

| **Study ID** | **Q1 - AN GROUP** | **Q2 - CONTROL SELECTION** | **Q3 - DEFINITION CONTROLS** | **Q4 - COMPARABILITY** | **Q5 - OUTCOME ASSESSMENT** | **Q6 - METHODS DESCRIPTION** | **Q7 - DATA PRESENTATION** | **Q8 - CONCLUSIONS** | **Q9 - LIMITATIONS** | | **TOTAL** | | **Risk of Bias** | |
| --- | --- | --- | --- | --- | --- | --- | --- | --- | --- | --- | --- | --- | --- | --- |
| Abell et al. (1987) | * |  | * | * | * | * | * | * |  | | 7 | | Low | |
| Bar et al. (2006) | * |  | * | ** | * | * | * | * | * | | 9 | | Low | |
| Bartak et al. (2004) | * |  | * | * | * | * | * | * |  | | 7 | | Low | |
| Billeci et al. (2015) | * | * |  | ** | * | * | * | * | * | | 9 | | Low | |
| Billeci et al. (2019) | * | * |  | ** | * | * | * | * | * | | 9 | | Low | |
| Bomba et al. (2014) |  |  | * | ** | * | * | * | * | * | | 8 | | Low | |
| Calloway et al. (1983) |  |  |  | * | * |  | * | * |  | | 4 | | Moderate | |
| Casu et al. (2002) |  |  |  | * |  | * | * | * |  | | 4 | | Moderate | |
| D'Andrea et al. (2008) |  |  |  | * | * | * | * | * |  | | 5 | | Moderate | |
| DeRosa et al. (1983) |  |  |  | * |  |  | * | * |  | | 3 | | High | |
| Dostalova et al. (2007) | * |  | * | * | * | * | * | * |  | | 7 | | Low | |
| Galetta et al. (2003) | * |  | * | * | * | * | * | * |  | | 7 | | Low | |
| Green et al. (2020) | * | * | * | * | * | * | * | * | * | | 9 | | Low | |
| Gross et al. (1979) |  |  | * | ** | * |  | * | * |  | | 6 | | Moderate | |
| Ishizawa et al. (2008) | * | * | * | * | * | * | * | * | * | | 9 | | Low | |
| Kaye et al. (1990) |  |  |  | * | * |  | * | * |  | | 4 | | High | |
| Kaye et al. (1985) |  |  |  |  | * |  | * | * | * | | 4 | | Moderate | |
| Kollai et al. (1994) | * |  |  | * | * | * |  | * | * | | 6 | | Moderate | |
| Koschke et al. (2010) | * | * | * | ** | * | * | * | * |  | | 9 | | Low | |
| Kreipe et al. (1994) |  |  |  | ** | * |  | * | * | * | | 6 | | Moderate | |
| Lachish et al. (2009) | * |  | * | ** | * | * | * | * | * | | 9 | | Low | |
| Lechin et al. (2010) | * |  | * | * | * | * | * | * |  | | 7 | | Low | |
| Leonard et al. (1998) |  |  |  | * | * | * | * | * | * | | 6 | | Moderate | |
| Lesem et al. (1989) |  |  | * | * | * | * | * | * |  | | 6 | | Moderate | |
| Lonigro et al. (2019) | * | * | * | * | * | * | * | * | * | | 9 | | Low | |
| Luck et al. (1983) |  |  |  | * | * | * | * | * |  | | 5 | | Moderate | |
| Lutz et al. (1998) | * | * | * | ** | * | * | * | * | * | | 10 | | Low | |
| Mazurak et al. (2011) | * | * | * | ** | * | * | * | * | * | | 10 | | Low | |
| Melanson et al. (2004) |  |  | * | * | * | * | * | * |  | | 6 | | Moderate | |
| Murialdo et al. (2007) |  |  |  | * | * | * | * | * |  | | 5 | | Moderate | |
| Nakai et al. (2015) | * | * | * | * | * | * | * | * | * | | 9 | | Low | |
| Nedvidkova et al. (2004) | * |  | * | * | * | * | * | * |  | | 7 | | Low | |
| Palomba et al. (2017) | * |  |  | * | * | * | * | * | * | | 7 | | Low | |
| Petretta et al. (1997) | * |  | * | ** | * | * | * | * |  | | 8 | | Low | |
| Pirke et al. (1992) |  |  | * | ** | * |  | * |  |  | | 5 | | Moderate | |
| Platisa et al. (2006) |  |  |  | ** | * | * | * | * | * | | 7 | | Low | |
| Rechlin et al. (1998) | * |  |  | ** | * | * |  | * |  | | 6 | | Moderate | |
| Riederer et al. (1982) |  |  |  | * | * |  | * | * |  | | 4 | | Moderate | |
| Roche et al. (2004) | * |  |  | * | * | * | * | * |  | | 6 | | Moderate | |
| Rommel et al. (2015) | * | * | * | * | * | * | * | * | * | | 9 | | Low | |
| Russell et al. (2008) |  |  | * | * | * |  | * | * |  | | 5 | | Moderate | |
| Takimoto et al. (2014) | * |  |  | * | * | * | * | * | * | | 7 | | Low | |
| Tonhajzerova et al. (2020) | * |  | * | * | * | * | * | * | * | | 8 | | Low | |
| VanBinsbergen et al. (1991) | * | * | * | ** | * | * | * | * |  | | 9 | | Low | |
| Vigo et al. (2008) | * |  | * | ** | * | * | * | * | * | | 9 | | Low | |
| Wu et al. (2004) | * |  | * | * | * | * | * | * |  | | 7 | | Low | |
| Risk of bias in studies was assessed by using a modified version of the Newcastle-Ottawa Quality Assessment Scale. | | | | | | | | | |  | |  | |  |
| The studies were scored out of 10. | |  |  |  |  |  |  |  |  | |  | |  | |
| 7 or more points was considered to be at low risk of bias, studies that scored 4-6 points to be at moderate risk, and those with less than 4 points to be at high risk of bias | | | | | | | | | | | | | | |
