# Supplementary material for: Autonomic Nervous System Function in Anorexia Nervosa: A Systematic Review
Source: Front Neurosci. 2021 Jun 28;15:682208. doi: 10.3389/fnins.2021.682208 (PMC8273292; doi:10.3389/fnins.2021.682208)
Supplement: Supplementary file 5 [file Data_Sheet_5.docx]

**Appendix 5. Heart rate variability risk of bias/quality assessment**

| **Study ID** | **Q1 - Diagnosis** | **Q2 - Recruitment** | **Q3 - Inclusion criteria** | **Q4 - Severity** | **Q5 - Software** | **Q6 - Collection** | **Q7 - Analysis** | **Q8 - Calculation** | **Q9 - Outcome** | **Q10 - Confounders assessed** | **Q11 - Confounder adjustment** | **Total** |
| --- | --- | --- | --- | --- | --- | --- | --- | --- | --- | --- | --- | --- |
| Bar et al. (2006) | 2 | 1 | 2 | 2 | 0 | 1 | 1 | 1 | 0 | 2 | 0 | 12 |
| Billeci et al. (2015) | 1 | 1 | 1 | 2 | 1 | 0 | 1 | 2 | 2 | 2 | 1 | 14 |
| Billeci et al. (2019) | 1 | 1 | 2 | 2 | 2 | 2 | 2 | 2 | 2 | 2 | 1 | 19 |
| Bomba et al. (2014) | 2 | 1 | 2 | 1 | 1 | 2 | 2 | 2 | 2 | 2 | 2 | 19 |
| Casu et al. (2002) | 1 | 0 | 1 | 2 | 0 | 0 | 0 | 0 | 0 | 2 | 1 | 7 |
| Galetta et al. (2003) | 1 | 1 | 1 | 2 | 2 | 0 | 2 | 1 | 2 | 1 | 1 | 14 |
| Green et al. (2020) | 1 | 1 | 1 | 0 | 2 | 2 | 2 | 2 | 2 | 0 | 0 | 13 |
| Ishizawa et al. (2008) | 2 | 2 | 2 | 2 | 2 | 2 | 1 | 1 | 2 | 2 | 1 | 19 |
| Kollai et al. (1994) | 1 | 0 | 1 | 0 | 0 | 2 | 1 | 1 | 1 | 1 | 0 | 8 |
| Koschke et al. (2010) | 1 | 1 | 2 | 2 | 2 | 2 | 1 | 2 | 2 | 2 | 2 | 19 |
| Kreipe et al. (1994) | 1 | 0 | 0 | 0 | 2 | 1 | 2 | 2 | 2 | 0 | 0 | 10 |
| Lachish et al. (2009) | 2 | 2 | 2 | 2 | 1 | 0 | 1 | 2 | 2 | 2 | 1 | 17 |
| Lonigro et al. (2019) | 1 | 2 | 2 | 1 | 2 | 1 | 0 | 0 | 2 | 2 | 0 | 13 |
| Lutz et al. (2019) | 1 | 2 | 2 | 2 | 2 | 0 | 1 | 2 | 1 | 1 | 1 | 15 |
| Mazurak et al. (2011) | 2 | 2 | 1 | 1 | 2 | 2 | 2 | 2 | 2 | 1 | 1 | 18 |
| Melanson et al. (2004) | 1 | 0 | 0 | 1 | 2 | 2 | 2 | 2 | 2 | 0 | 0 | 12 |
| Murialdo et al. (2007) | 1 | 0 | 0 | 2 | 0 | 2 | 0 | 1 | 2 | 1 | 1 | 10 |
| Nakai et al. (2015) | 1 | 2 | 2 | 2 | 2 | 2 | 2 | 2 | 2 | 2 | 2 | 21 |
| Petretta et al. (1997) | 2 | 2 | 1 | 1 | 2 | 2 | 2 | 2 | 2 | 2 | 1 | 19 |
| Platisa et al. (2006) | 1 | 0 | 1 | 2 | 2 | 2 | 2 | 2 | 2 | 1 | 1 | 16 |
| Rechlin et al. (1998) | 1 | 0 | 2 | 0 | 2 | 2 | 2 | 2 | 2 | 2 | 0 | 15 |
| Roche et al. (2004) | 1 | 1 | 1 | 1 | 2 | 0 | 2 | 2 | 2 | 1 | 0 | 13 |
| Rommel et al. (2015) | 2 | 2 | 2 | 2 | 0 | 2 | 1 | 1 | 0 | 1 | 1 | 14 |
| Russell et al. (2008) | 2 | 0 | 0 | 1 | 2 | 0 | 0 | 0 | 2 | 1 | 1 | 9 |
| Tonhajzerova et al. (2020) | 2 | 2 | 2 | 2 | 1 | 2 | 2 | 2 | 2 | 2 | 2 | 21 |
| Vigo et al. (2008) | 2 | 1 | 2 | 2 | 1 | 1 | 2 | 2 | 2 | 1 | 1 | 17 |
| Wu et al. (2004) | 1 | 0 | 1 | 1 | 2 | 2 | 2 | 1 | 2 | 1 | 0 | 13 |
